# Trace-stimuli-triggered controlled degradation for hydrogel adhesives

**DOI:** 10.1093/nsr/nwaf498

**Published:** 2025-11-19

**Authors:** Siming Li, Zilong Han, Weiqi Xu, Mingzhi Su, Yuchen Lu, Yao Shen, Yan Mu, Heng Zhu, Xiaohui Song, Panpan Ye, Ke Yao, Wei Yang, Shaoxing Qu

**Affiliations:** Zhejiang University, Eye Center of Second Affiliated Hospital, School of Medicine, Hangzhou 310009, China; State Key Laboratory of Fluid Power & Mechatronic System, Key Laboratory of Soft Machines and Smart Devices of Zhejiang Province, Center for X-Mechanics, and Department of Engineering Mechanics, Zhejiang University, Hangzhou 310027, China; Zhejiang University, Eye Center of Second Affiliated Hospital, School of Medicine, Hangzhou 310009, China; State Key Laboratory of Fluid Power & Mechatronic System, Key Laboratory of Soft Machines and Smart Devices of Zhejiang Province, Center for X-Mechanics, and Department of Engineering Mechanics, Zhejiang University, Hangzhou 310027, China; Department of Hepatic Surgery, Shanghai Cancer Center, Fudan University, Shanghai 200032, China; Zhejiang University, Eye Center of Second Affiliated Hospital, School of Medicine, Hangzhou 310009, China; State Key Laboratory of Fluid Power & Mechatronic System, Key Laboratory of Soft Machines and Smart Devices of Zhejiang Province, Center for X-Mechanics, and Department of Engineering Mechanics, Zhejiang University, Hangzhou 310027, China; Zhejiang Key Laboratory of Clean Energy Conversion and Utilization, Science and Education Integration College of Energy and Carbon Neutralization, Zhejiang University of Technology, Hangzhou 310014, China; State Key Laboratory of Fluid Power & Mechatronic System, Key Laboratory of Soft Machines and Smart Devices of Zhejiang Province, Center for X-Mechanics, and Department of Engineering Mechanics, Zhejiang University, Hangzhou 310027, China; State Key Laboratory of Fluid Power & Mechatronic System, Key Laboratory of Soft Machines and Smart Devices of Zhejiang Province, Center for X-Mechanics, and Department of Engineering Mechanics, Zhejiang University, Hangzhou 310027, China; Zhejiang University, Eye Center of Second Affiliated Hospital, School of Medicine, Hangzhou 310009, China; Zhejiang University, Eye Center of Second Affiliated Hospital, School of Medicine, Hangzhou 310009, China; Zhejiang University, Eye Center of Second Affiliated Hospital, School of Medicine, Hangzhou 310009, China; State Key Laboratory of Fluid Power & Mechatronic System, Key Laboratory of Soft Machines and Smart Devices of Zhejiang Province, Center for X-Mechanics, and Department of Engineering Mechanics, Zhejiang University, Hangzhou 310027, China; Zhejiang University, Eye Center of Second Affiliated Hospital, School of Medicine, Hangzhou 310009, China; State Key Laboratory of Fluid Power & Mechatronic System, Key Laboratory of Soft Machines and Smart Devices of Zhejiang Province, Center for X-Mechanics, and Department of Engineering Mechanics, Zhejiang University, Hangzhou 310027, China

**Keywords:** hydrogel, tissue adhesives, degradation, low activation threshold

## Abstract

Biodegradable hydrogels are promising for tissue adhesives and implantable coatings, but are often limited by harsh degradation triggers and uncontrolled breakdown. Here, we present a biodegradable hydrogel design strategy that leverages a redox-responsive crosslinker with a low activation threshold, enabling spatiotemporally controlled degradation in response to trace levels of reactive oxygen species (ROS). The hydrogel undergoes rapid and complete degradation, even under low redox stress, disassembling entirely within 2 h under 0.1% (w/w) ROS, and 24 h at concentrations as low as 0.0001% (w/w) (37°C). Functioning as a tissue adhesive, the hydrogel forms bonds within 5 s, maintains strong wet adhesion (200 J/m²), and exhibits excellent sealing performance in both *in vitro* and *in vivo* models, with complete degradation under physiological ROS concentrations [∼0.0000001% (w/w)] occurring over 3 weeks—well aligned with the natural wound healing process. Notably, initial mild degradation triggers chain growth, which reinforces wet adhesion by actively compensating for swelling-induced interfacial weakening. The strategy demonstrates remarkable generality and biocompatibility, facilitating the clinical application of biodegradable materials and minimizing the risk of synthetic residue and contamination.

## Teaser

A biodegradable hydrogel strategy is proposed, leveraging a redox-responsive crosslinker with low activation threshold to achieve spatiotemporally controlled degradation of the hydrogel via trace reactive oxygen species (ROS) and complete *in vivo* degradation within 3 weeks.

## INTRODUCTION

Functional biomaterials have been essential in advancing tissue engineering, driving breakthroughs in tissue repair, augmentation and regenerative medicine [1–3]. The practical application of these materials, however, is constrained by demanding environmental conditions, limiting their broader clinical utility [4]. These challenges are particularly evident in emerging fields such as tissue adhesives, medical implants and brain–machine interfaces, where current solutions frequently underperform in physiological environments. For instance, residual tissue adhesives can impair healing [5], while unremoved implants can lead to fibrosis [6]. Tissue adhesives, a subset of temporary functional materials, are widely used in surgical procedures, functioning as hemostatic agents, tissue sealants or localized delivery vehicles [7]. Their intended function is to bind tissues or prevent leakage during the healing process, with an ideal destiny of complete post-recovery disappearance. A major obstacle to the effective use of temporary functional materials lies in the difficulty of controlled degradation [8]. Most current tissue adhesives, including cyanoacrylate, fibrin sealants and hydrogels, encounter issues with severe degradation requirements or prolonged, uncontrollable degradation timelines, which conflict with natural healing processes [9]. Hydrogels, in particular, stand out as promising tissue adhesives owing to their excellent biocompatibility and mechanical compliance with surrounding tissues [7,10]. Yet, achieving reliable and controlled degradation under physiological conditions remains a significant hurdle.

Tissue adhesives that degrade rather slowly or not at all may impair the normal growth and remodeling of new tissue, delaying the healing process and increasing the risk of wound infection. Conversely, excessively rapid degradation fails to provide sufficient support and protection to the wound for an adequate duration, leading to suboptimal healing outcomes [11]. To achieve precise control over the degradation kinetics and mechanisms of wound-healing adaptive hydrogels, while concurrently maintaining excellent biocompatibility and mechanical properties, strategies are focused on several key avenues, including the tailored molecular architecture design [12,13], the integration of nanotechnology [14] and the optimization of crosslinked network structures [15,16]. Central to these strategies is the utilization of endogenous biological stimuli, typically involving three main mechanisms: hydrolysis [8,17], redox degradation [18,19] and enzymatic degradation [20]. Nevertheless, the high-water content *in vivo* makes hydrolysis difficult to control [21]. Furthermore, natural polysaccharides, such as dextran or alginate, lack corresponding endogenous enzymes for degradation in the human body [22]. Redox degradation generally originates from reactive oxygen species (ROS: ·OH, O_2_^·^^−, 1^O_2_, H_2_O_2_), which are produced in both normal physiological processes and pathological states and are playing various roles within cells [23]. By contrast, ROS-stimulated degradation can achieve more precise spatiotemporal control, enabling intelligent responsiveness. Wound healing typically takes 1 to 3 weeks, necessitating the degradation of adhesives to align with this timeframe [24]. On the one hand, high covalent bond cleavage thresholds require elevated stress concentrations. For instance, the thioketal bond (bond energy: 272 kJ/mol) is difficult to degrade at physiological concentrations [25,26]. Similarly, other redox-sensitive bonds, such as disulfide [12] and thioether [27], face the same challenge. On the other hand, chemical bonds with excessively low bond energy, such as the ditelluride bond (bond energy: 125 kJ/mol), tend to undergo premature and excessive degradation during the critical stages of wound healing due to their inherent instability, failing to provide sustained mechanical support for tissues [28]. These limitations greatly restrict the practical application of them in tissue adhesives [9,29,30]. The critical challenge lies in achieving controlled degradation in response to trace stimuli, ensuring the timely removal of hydrogel adhesives after fulfilling their role in tissue adhesion. Moreover, biocompatibility must be maintained throughout the degradation process to align with the physiological demands of wound healing, reducing the need for secondary medical interventions [16,31].

**Figure 1.**
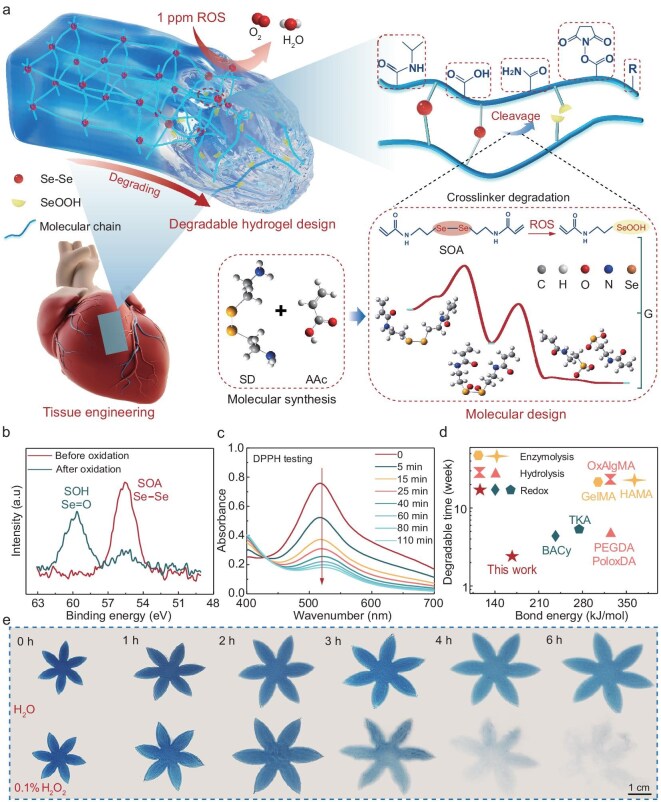
Design and degradation mechanism of hydrogels controlled by trace ROS. (a) Schematic illustration of the strategy and degradation mechanism of hydrogel. SD, selenocystamine dihydrochloride. (b) XPS spectra of the low-redox-stress-threshold SOA crosslinker before and after oxidation. (c) UV–vis spectra of DPPH/SOA solution over 110 min. (d) *In vivo* degradation times of hydrogels prepared with different crosslinkers. (e) Macroscopic degradation comparison of the degradable hydrogels in pure water and 0.1% (w/w) H_2_O_2_ within 6 h (25°C).

Here, we propose a biodegradable hydrogel design strategy that leverages a novel universal hydrogel crosslinker [*N,N*′-(diselenediylbis(ethane-2,1-diyl))diacrylamide; SOA], featuring a low redox stress threshold. This enables the crosslinking of diverse monomers to form multifunctional hydrogels capable of controlled degradation under trace levels of ROS (Fig. 1a). The crosslinkers function as degradation switches at physiologically tolerable concentrations, while the combination of high- and low-threshold redox stress crosslinkers finely tunes the degradation kinetics of the hydrogel on a macroscopic scale. Biodegradable hydrogel adhesives engineered via this strategy exhibit rapid and robust tissue adhesion and undergo complete degradation within 3 weeks *in vivo*. Notably, early-stage mild degradation triggers chain growth, accelerating water diffusion to mitigate interface plasticization, thereby stabilizing adhesion to wet/bleeding tissues synergistically. Our findings present a precise, trace-stimuli-responsive degradation strategy for hydrogels, offering a solution for the safe and controlled removal of temporary functional materials and enhancing the biocompatibility and clinical applicability of degradable hydrogels.

## RESULTS

### Design and mechanisms of biodegradable hydrogels

Our strategy utilizes a low-redox-stress-threshold diselenide-based crosslinker (172 kJ/mol) to achieve degradation under trace-level ROS, since these bonds are more susceptible to electron loss during the reaction. Before synthesizing the crosslinker, we mapped the degradation pathway, including the transition states, intermediates and final products (Fig. S1a). The thermodynamic process of SOA crosslinker degradation progression revealed the intrinsic mechanism and kinetic parameters of the chemical bond cleavage (Fig. S1b–S1e and Table S1). Under the action of H_2_O_2_, the diselenide bond undergoes two transition states before being oxidized to stable selenic acid, with ΔG < 0 indicating the spontaneity of the reaction.

We prepare the SOA crosslinker via the condensation reaction of carboxylic acid and amine. Specifically, under the influence of the coupling reagent benzotriazol-1-yl-oxytripyrrolidinophosphonium hexafluorophosphate (PyBOP) and the catalyst *N,N*-diisopropylethylamine (DIPEA), the carboxyl group is activated *in situ* to form an active intermediate, which then undergoes a nucleophilic substitution reaction with the amine, efficiently forming an amide bond. This reaction is completed under mild conditions (room temperature, 30 min), with water being the only byproduct. Compared to the traditional acyl chloride and amine reactions, the reaction taken by this work yields high productivity (90%) and high-water solubility (Fig. S2a). To characterize the structure of the synthesized crosslinker and its degradation products, X-ray photoelectron spectroscopy (XPS) (Fig. 1b and Fig. S2b) and Fourier transform infrared spectroscopy (FTIR) (Fig. S2c) were employed. According to the XPS results, the 3d^5^ binding energy of the Se atoms of the SOA crosslinker increased from 55.7 to 59.0 eV after oxidation, approximating the valence state of the selenic acid group [32]. FTIR spectra evidenced the disappearance of the Se–Se peak at 797 cm⁻^1^ post-oxidation, and the emergence of a new peak near 878 cm⁻^1^, indicative of Se=O vibrations [33]. The antioxidant capacity of the SOA crosslinker was evaluated using the 2,2-diphenyl-1-picrylhydrazyl (DPPH) assay [34]. with UV–vis spectroscopy illustrating a progressive decrease in DPPH absorbance over time in the DPPH/SOA mixture, confirming persistent radical scavenging (Fig. 1c). At 120 min, the DPPH/SOA solution visibly paled relative to the DPPH ethanol solution control, with roughly 76% of DPPH neutralized by 4 mg/mL SOA (Fig. S2d). The findings underscore the antioxidant capacity of the SOA crosslinker, suggesting its potential to counteract oxidative stress in biological systems. The mass spectrum and ^1^H NMR further elucidate the structure and characteristics of the SOA crosslinker (Figs S3 and S4).

The key to degradation under trace-level stimulation conditions lies in the sensitivity of bond cleavage, that is, the low redox stress threshold of covalent bonds. By comparing crosslinkers with different bond energies [8,35], we found that the low-redox-stress-threshold crosslinker exhibits the most rapid and controllable degradation kinetics under trace-level stimulation *in vivo* (Fig. 1d). In addition, the macroscopic degradation scenario of hydrogels was visually assessed in both pure water and a 0.1% (w/w) H_2_O_2_ environment (25°C) (Fig. 1e). In pure water, the hydrogel progressively swelled yet maintained its structural integrity within 6 h, whereas the hydrogel underwent significant architectural disintegration in 0.1% (w/w) H_2_O_2_ after approximately 3 h, and completely disappeared after 6 h, exemplifying its rapid degradation under low-stimulation conditions (Movie S1).

### *In vitro* degradation performance

**Figure 2.**
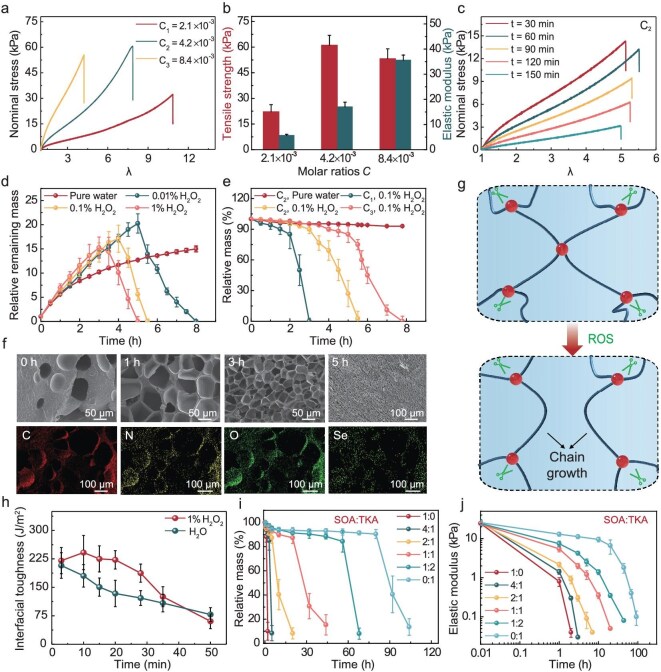
Degradation performance of trace-level ROS-degradable hydrogels. (a and b) The impact of crosslinker content on the mechanical properties of as-prepared degradable hydrogels, including nominal strain–nominal stress curves (a) and the elastic modulus and tensile strength (b). (c) The effects of degradation on the mechanical properties of hydrogels in 0.1% (w/w) H_2_O_2_ within 150 min (25°C). (d) The relative remaining mass of the hydrogel at different times varies with the concentration of H_2_O_2_ (25°C). (e) Hydrogels with lower concentrations of crosslinkers exhibit easier mass loss under identical time and stimulation conditions (25°C). (f) SEM and EDS images of degradable hydrogels over time. (g) Microscopic schematic diagram of chain growth caused by crosslinker breakage under ROS stimulation. (h) The variation in interfacial toughness of fast-degrading and non-degrading hydrogels over time in a humid environment (37°C). (i and j) The mass loss (i) and elastic modulus (j) changes of SOA/TKA mixed crosslinker hydrogels over time by using 0.1% (w/w) H_2_O_2_ as stimulus (37°C). Unless otherwise specified, all experiments are based on a crosslinker-to-monomer molar ratio of C_2_ as an example. Values represent the mean and standard deviation (*n* = 3–5).

The redox stress threshold and content of the crosslinker directly influence the mechanical properties and degradation kinetics of the hydrogel. To validate the versatility of the low-redox-stress-threshold SOA crosslinker, a variety of functional monomers such as acrylamide (Aam), acrylic acid (Aac) and *N*-isopropylacrylamide (NIPAm) were employed to generate rapidly degradable hydrogels. These hydrogels can be fabricated through either thermal or photoinitiation methods, exhibiting outstanding degradation properties (Figs S5 and S6). More intricate geometries can be fabricated via digital light processing (DLP) 3D printing to meet the practical requisites of biomedical engineering, such as defect tissue filling material (Fig. S7). Herein, polyacrylamide (PAAm) hydrogel was utilized, where crosslinker concentration significantly influenced the structural stability and mechanical properties of the hydrogel (Fig. 2a and b). As the amount of crosslinker increases, the crosslinking density enhances the network stability and structural strength, thereby improving the strength and modulus of the hydrogel. Excessive crosslinking, however, may cause brittle fracture, resulting in a decrease in strength. In contrast, the modulus of the hydrogel shows a gradient decrease due to the combined effect of crosslinker degradation and swelling in the hydrogel. Early-stage crosslinker degradation leads to chain growth, resulting in a higher elongation at the break in the degraded hydrogel, which helps to maintain its resistance to fracture work. In the later stages of degradation, swelling further accelerates this deconstruction, ultimately leading to the loss of mechanical properties (Fig. 2c and Fig. S8a). The effect of ROS concentration (Fig. 2d and Fig. S8b) and crosslinker concentration (Fig. 2e and Fig. S8c) on hydrogel degradation was investigated (at 25°C). Scanning electron microscopy (SEM) and energy-dispersive spectroscopy (EDS) (Fig. 2f) revealed that the crosslinker was uniformly distributed within the hydrogel. As degradation progresses, the hydrogel network loses its structural integrity. Following freeze-drying pretreatment, the network collapsed, resulting in a denser structure. Specifically, the Se–Se bonds underwent cleavage under ROS stimulation, leading to network fracture while simultaneously inducing chain growth and swelling (Fig. 2g). For pure swelling hydrogels, the swelling causes a continuous decrease in the interfacial toughness [36]. However, the degradation-mediated chain growth promotes water diffusion from the interface to the bulk (Fig. S9) [37–39], weakening the plasticizing effect of free water at the interface while enhancing chain flow to strengthen interface–bulk interactions [40,41]. These combined effects reinforce the early adhesion performance during *in vitro* simulations (Fig. 2h). More importantly, this synergistic mechanism can be selectively regulated according to the duration of the wound healing cycle, allowing the hydrogel to meet the requirements of wounds with different healing durations, and maintain effective adhesion during critical stages.

We also regulated hydrogel degradation kinetics by incorporating two crosslinkers with distinct degradation rates—*N,N*′-((propane-2,2-diylbis(sulfanediyl))bis(ethane-2,1-diyl) diacrylamide (TKA), a high-redox-stress-threshold crosslinker (Fig. S10), and SOA, a fast-degrading crosslinker. The high-threshold thioketal bonds in TKA ensure gradual degradation, while rapid cleavage of SOA initiates early-stage chain reorganization. Adjusting the TKA/SOA ratio allowed precise control over swelling, mechanical properties and mass loss, with degradation times ranging from 2 to 120 h at 37°C (Fig. 2i and j, and Fig. S11). Additionally, degradation kinetics could be fine-tuned through single or combined stimuli (ROS, water, enzyme, pH etc.). This approach allows stage-specific degradation—rapid early-phase crosslinker cleavage to reinforce adhesion through chain remodeling, followed by stable late-phase degradation to meet diverse application needs.

### *Ex vivo* adhesion performance

**Figure 3.**
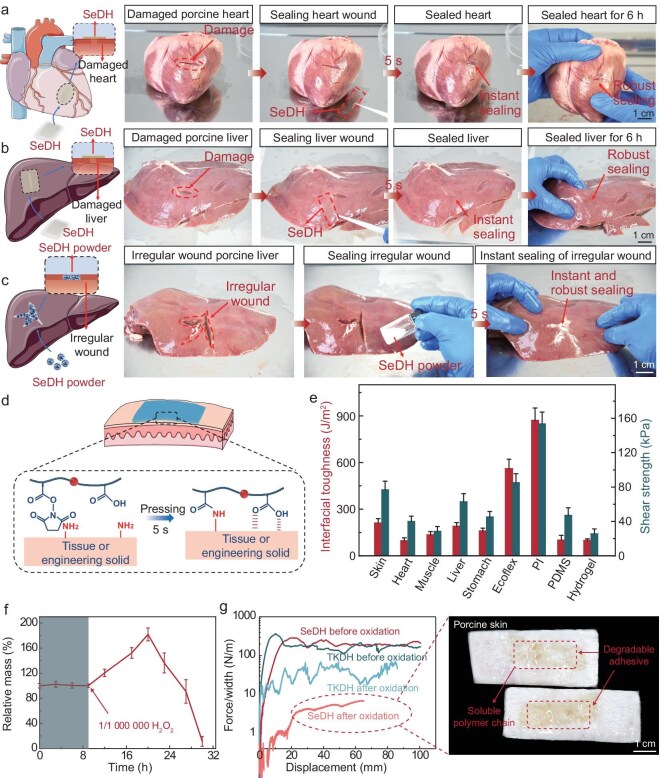
*Ex vivo* adhesive properties and prospective applications of trace ROS-degradable hydrogel adhesive (SeDH). (a and b) Sealing of a wound on an *ex vivo* porcine heart (a) and porcine liver (b) by SeDH. (c) Sealing of an irregular wound on an *ex vivo* porcine liver using SeDH powder. (d) Schematic illustration of the adhesion mechanism of SeDH with wet tissue or engineered solids. (e) Interfacial toughness and shear strength between various tissues and engineering solids adhered by the SeDH. (f) Relative mass change of SeDH degrading in 1 ppm H_2_O_2_ (37°C). (g) Adhesion performance of TKA or SOA crosslinked hydrogels bonding porcine skin for 6 h using 0.1% (w/w) H_2_O_2_ as stimulus (37°C). Values represent the mean and standard deviation (*n* = 3–5).

An optimal temporary tissue adhesive must offer essential mechanical assistance without impeding natural healing progression in the early stages, and subsequently degrade to permit the nascent tissue to assume the mechanical functions [42]. Therefore, we devised a low-redox-stress-threshold SOA-crosslinked degradable poly(acrylic acid) (PAAc)/PAAc-*N*-hydroxysuccinimide (PAAc-NHS) hydrogel adhesive (SeDH) (Fig. S12a–S12c) [43]. The fully swollen SeDH maintains high extensibility and mechanical dissipation mechanisms, manifesting a fracture toughness of approximately 460 J/m², thereby mechanically harmonizing with tissue demands (Fig. S12d and S12e) [44,45]. We evaluated the potential application of SeDH adhesive in tissue adhesion using *ex vivo* tissue models. The SeDH adhesive could effectively seal wounds on the heart (Fig. 3a, S13a and Movie S2) and liver (Fig. 3b, Fig. S13b and Movie S3) within 5 s and maintained robust sealing in a moist environment for an extended duration of 6 h. To accommodate irregular wound geometries, particularly on tissues like the liver (Fig. 3c and Movie S4), the SeDH was innovatively transformed into a powdery form, evidencing an impressive interfacial toughness of around 200 J/m² (Fig. S14 and Movie S5). This strong adhesion came from the intermolecular forces and covalent interactions between PAAc/PAAc-NHS and wet tissues (Fig. 3d and Fig. S15) [46]. Next, a comprehensive assessment of the SeDH adhesive performance across various wet tissues was conducted, including skin, heart, muscle, liver and stomach (Fig. 3e, Fig. S16a–S16c and S16g, and Movies S6 and S7), with interfacial toughness and shear strength of 210 ± 29.3 J/m^2^ and 76.8±10 kPa for skin, 96.5 ± 19.4 J/m^2^ and 39.89 ± 6.3 kPa for heart, 135 ± 20.7 J/m^2^ and 28.4 ± 5.7 kPa for muscle, 190 ± 23.5 J/m^2^ and 62.9 ± 9.4 kPa for liver, and 160.3±18 J/m^2^ and 45.3 ± 6.2 kPa for stomach. Beyond biological tissues, the adhesive also showed commendable adhesion properties with a range of amine-functionalized engineered solids (Fig. S17), such as Ecoflex, polyimide (PI), polydimethylsiloxane (PDMS) and hydrogel (Fig. 3e, Fig. S16d–S16f and S16g), achieving interfacial toughness and shear strength of 560.4 ± 60.7 J/m^2^ and 85 ± 10.5 kPa for Ecoflex, 870.6 ± 80.34 J/m^2^ and 153.4 ± 13.8 kPa for PI, 101.2 ± 30.7 J/m^2^ and 47 ± 8.9 kPa for PDMS, and 99.5±10 J/m^2^ and 25.5 ± 5.8 kPa for hydrogel.

In addition, we simulated the internal oxidative environment of the human body *in vitro* to study the degradation of the prepared SeDH. The concentration of ROS in normal human tissues is maintained at approximately 10^−8^ M, while in inflamed tissues or tumors, the ROS concentration is several dozen to several thousand times higher than that in normal tissues [47]. In simulation experiments *in vitro*, the mass significantly increased due to the cleavage of the crosslinker and the swelling of the hydrogel, followed by rapid degradation of the SeDH adhesive, upon triggering the redox stress-induced degradation switch (1 ppm of H_2_O_2_) (Fig. 3f). The rapid hydration and degradation underpinned the controllability of its degradation strategy. The mechanical properties of TKA- and SOA-crosslinked hydrogels were compared by using skin tissue (Fig. 3g and Fig. S18) and PI (Fig. S19) engineered solids before and after oxidation [0.1% (w/w) H_2_O_2_ as stimulus], respectively. After 6 h, SOA-crosslinked hydrogels exhibited a significant reduction in toughness post-oxidation, indicating their suitability for applications requiring rapid degradation and detachment. Contrarily, there was no debonding caused by rapid degradation under the same conditions for TKA-crosslinked hydrogels, which might lead to issues such as infection or inflammatory responses due to residual adhesive. The divergent degradation behaviors of these two crosslinkers demonstrated the practical benefits of the wound-healing adaptive ROS-degradable hydrogel strategy.

### *In vivo* biodegradability and potential applications

**Figure 4.**
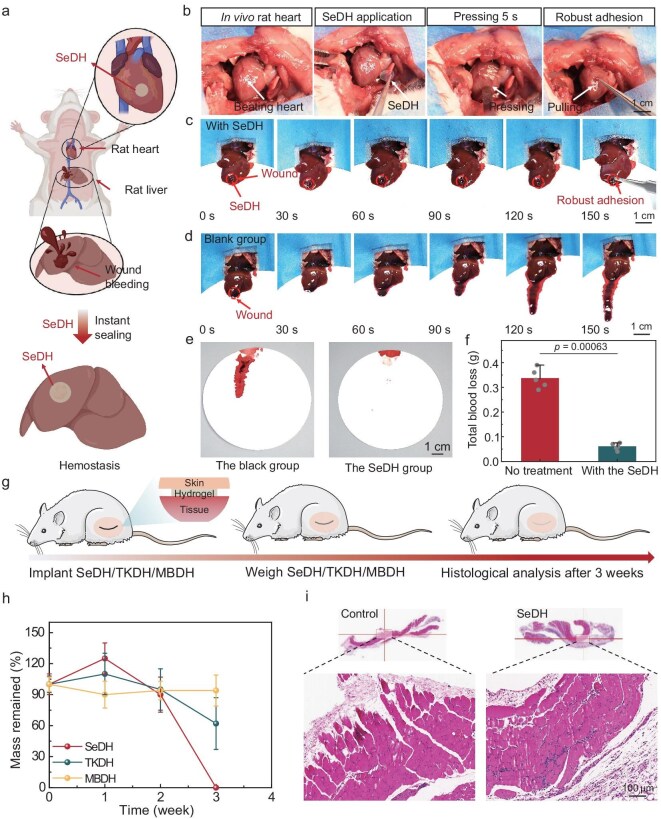
*In vivo* adhesion, biocompatibility and biodegradability of the SeDH. (a) Schematic illustration of dynamic heart adhesion and liver wound hemostasis in a rat model using SeDH. (b) Adhesion of an SeDH on a beating rat heart *in vivo*. (c and d) Schematic illustration of hemostasis in a damaged liver in the group with SeDH (c) and the blank control group (d). (e) Blood stains on filter paper surfaces at 150 s in the blank control group and the SeDH group. (f) Total blood loss mass in the blank control group and the SeDH group. (g) Schematic illustrations for biocompatibility and biodegradability of implants (SeDH/TKDH/MBDH) in the subcutaneous tissue of the lateral back of mice. (h) Comparison of the *in vivo* degradation degrees of different hydrogels (SeDH/TKDH/MBDH). (i) Representative histological images stained with H&E for assessing the biocompatibility of subcutaneously implanted hydrogels based on SeDH after 3 weeks. Values represent the mean and standard deviation (*n* = 3–5).

We demonstrate the *in vivo* adhesion and degradation of the adhesive. To verify the adhesion performance of the SeDH adhesive on dynamic wet tissues, it was directly applied to the epicardial surface of a rat heart (Fig. 4a and b, and Movie S8). The adhesive swiftly formed a robust bond on the cardiac surface, maintaining secure adhesion despite the continuous mechanical stress of the beating heart. This feature is of paramount importance for interventions involving constantly mobile organs. Subsequently, the hemostatic efficacy of SeDH was investigated using a rat liver puncture wound model (Fig. 4a). Upon creating the wound, the adhesive was immediately applied to the blood-covered liver surface, with a non-intervention group serving as the control. In the treated group, minimal blood seepage was discernible on the filter paper placed underneath the liver section (Fig. 4c), contrasting starkly with the control group, where a conspicuous bleeding trail was evident (Fig. 4d). The bloodstains in the control group became more pronounced but remained negligible in the experimental group as time passed. The blood loss mass from the rat liver was measured after 150 s (Fig. 4e and f). The total mass in the SeDH group (0.06 g) was significantly less than that in the control group (0.33 g), attesting to its superior hemostatic performance. Notably, even when exposed to mild mechanical manipulation, such as gentle pushing, pulling or pressing, the adhesive film adhered steadfastly to the liver surface. The tenacious adhesive capability curtailed blood diffusion and reinforced wound sealing (Movie S9). In larger animals with higher blood flow (such as rabbits), the SeDH adhesive also demonstrated excellent adhesion and hemostatic properties (Figs S20 and S21). More importantly, as a high-efficiency hemostatic material, SeDH exhibited superior blood compatibility (Fig. S22). This property, in synergy with the aforementioned excellent *in vivo* adhesive performance and hemostatic efficacy, further underscores its potential for clinical applications.

For hydrogels intended for tissue repair, they must not only degrade appropriately but also foster a biocompatible microenvironment that supports cell migration, proliferation and tissue regeneration at the site of damage. Firstly, we co-cultured mouse embryonic fibroblasts (NIH-3T3 cells) with three different conditioned media [SeDH, TKA-crosslinked degradable PAAc/PAAc-NHS (TKDH) and MBAA-crosslinked degradable PAAc/PAAc-NHS (MBDH)] to evaluate the *in vitro* biocompatibility and cell behavior during the culture process. The cell viability assay (CCK-8) revealed that all experimental groups sustained cell viability above 80% compared to the untreated control, affirming the biocompatible nature of SeDH adhesives (Fig. S23a). Secondly, almost no dead cells (red staining) were observed in either the experimental or control groups in the live/dead staining, accompanied by significant cell proliferation over a 5-day period (Fig. S23b). These findings collectively suggest that SeDH positively influences cell migration and proliferation, thereby facilitating wound healing. In addition, we investigated the biocompatibility of SeDH after degradation [48]. There was no observable decrease in the *in vitro* viability of NIH-3T3 after 24-h culture (Fig. S24). The designed SeDH ultimately degraded into macromolecular chains that were rapidly absorbed and eliminated from the body (Figs S25 and S26) [49].

Finally, we evaluated the *in vivo* biocompatibility and biodegradability of SeDH in a subcutaneous implantation model in mice (Fig. 4g). Consistent with the *in vitro* degradation behavior [8], the SeDH adhesive underwent active and rapid degradation within 2–3 weeks upon activation of the redox stress switch. Conversely, TKDH and MBDH exhibited slow and non-degradable characteristics over the observation period (Fig. 4h and Movie S10). To investigate the physiological side effects associated with the biodegradation byproducts, histological analyses were performed on tissues adjacent to the implanted hydrogel samples (Fig. 4i). Histological evaluation showed that tissues in contact with SeDH did not exhibit significant inflammatory reactions after 3 weeks. In addition, SeDH exhibited superior bonding performance along with flexible and controllable degradability compared to existing tissue adhesives (Fig. S27 and Table S2). Overall, SeDH adhesives have been demonstrated to possess exceptional biocompatibility, supporting cellular activities conducive to tissue repair, alongside tunable degradability that aligns well with the healing timeline.

## CONCLUSION

Bioadhesives and implants that are non-degradable or degrade in an uncontrolled manner often exhibit poor adaptability and are prone to foreign body reactions, fibrosis and complications requiring surgical removal. However, existing degradation strategies still suffer from harsh activation conditions, uncontrollable degradation processes and toxic byproducts, limiting the clinical use of biodegradable materials. An ideal adhesive must ensure interface stability and exhibit safe and controllable degradation behavior. In this study, we report a low-redox-stress-threshold biodegradable hydrogel design strategy that leverages trace levels of ROS to enable swift and controllable degradation of biomaterials. Meanwhile, we demonstrate that initial mild chain growth is beneficial for mitigating interfacial plasticization, enhancing the wet adhesion effect. The bioadhesive prepared by this method has been preliminarily validated *in vitro*/*in vivo*, enhancing biocompatibility, spatiotemporal degradation control and clinical feasibility, providing a safer alternative for temporary biomedical implants.

In addition, the integration of low-redox-stress-threshold degradable crosslinkers with functional monomers enables the creation of biomaterials with controllable degradation, making them highly suitable for tissue adhesion, medical device coatings [50] and drug delivery applications (Fig. S28). Importantly, this approach extends beyond hydrogels and is adaptable to other polymer systems, including elastomers and rubbers. Beyond hydrogels, the strategy applies to degradable elastomers and rubbers, offering material platforms that respond to environmental or physiological triggers. Overall, the trace-stimuli-triggered degradation system offers a clinically viable pathway toward safe and programmable tissue adhesives, paving the way for next-generation solutions in wound healing and regenerative implants.

## METHODS

Detailed materials and methods are available in the online Supplementary data.

## ETHICAL STATEMENT

This study was performed in accordance with the recommendations in the Guide for the Care and Use of Laboratory Animals and relevant Chinese laws and regulations. All the animal assays were approved by the Institutional Animal Care and Use Committee of Zhejiang University (2024188) and Fudan University (2023-09-YJ-QJP-82).

## Supplementary Material

nwaf498_Supplemental_File
