# Supplementary material for: Trace-stimuli-triggered controlled degradation for hydrogel adhesives
Source: Natl Sci Rev. 2025 Nov 19;13(1):nwaf498. doi: 10.1093/nsr/nwaf498 (PMC12805828; doi:10.1093/nsr/nwaf498)
Supplement: nwaf498_Supplemental_File [file nwaf498_supplemental_file.pdf]

Supporting Information

Trace-stimuli-triggered controlled degradation for hydrogel adhesives

Siming Li[1,2], Zilong Han[1,2]*, Weiqi Xu[3], Mingzhi Su[1], Yuchen Lu[2], Yao Shen[4], Yan Mu[2], Heng Zhu[2], Xiaohui Song[1], Panpan Ye[1], Ke Yao[1], Wei Yang[2] & Shaoxing Qu[1,2]*

[1]Zhejiang University, Eye Center of Second Affiliated Hospital, School of Medicine, China.

[2]State Key Laboratory of Fluid Power & Mechatronic System, Key Laboratory of Soft Machines and Smart Devices of Zhejiang Province, Center for X-Mechanics, and Department of Engineering Mechanics, Zhejiang University, Hangzhou 310027, China.

[3]Department of Hepatic Surgery, Shanghai Cancer Center, Fudan University, Shanghai 200032, China.

[4]Zhejiang Key Laboratory of Clean Energy Conversion and Utilization, Science and Education Integration College of Energy and Carbon Neutralization, Zhejiang University of Technology, Hangzhou, 310014, China.

*__Corresponding authors__.

Email: hanzilong@zju.edu.cn (Z.H.); squ@zju.edu.cn (S.Q.).

The PDF file includes:

Experimental methods

Fig. S1 to S28

Table S1 and S2

References

Legends for movies S1 to S10

Other Supplementary Materials for this manuscript includes the following:

Movies S1 to S10

Experimental methods

Materials

Acrylamide (AAm), Acrylic acid (AAc), N-Isopropyl acrylamide (NIPAm), polyvinyl alcohol (PVA), 3-aminopropyltriethoxysilane (APTES), Acryloyl chloride, Dimethyl sulfoxide (DMSO), Poly(3,4-ethylenedioxythiophene)/poly(styrenesulfonate)(PEDOT: PSS), Ammonium persulfate (APS), N, N'-methylene diacrylamide (MBAA), 4,4'-Azobis(4-cyanovaleric acid) (ACVA), N, N-Dimethylformamide (DMF), and selenocystamine dihydrochloride (SD) were purchased from Aladdin (Shanghai, China). Polyacrylic acid (PAA) solution (M_w~100 kDa) purchased from Macklin (Shanghai). Acrylic acid N-hydroxysuccinimide ester (AAc-NHS ester) was purchased from TCI (Japan). Lithium Phenyl(2,4,6-trimethylbenzoyl) phosphinate (LAP) was purchased from EFL (Suzhou, China). Hydrophilic cyanine 7 (CY7), 2-[2-(2-aminoethylsulfanyl) propan-2-ylsulfanyl] ethanamine (TK-NH_2), N, N-Diisopropylethylamine (DIPEA), Benzotriazol-1-yl-oxytripyrrolidinophosphonium hexafluorophosphate (PyBOP), Dichloromethane (DCM), and Methanol (MeOH) were supported by Yusi Pharmaceutical Technology Co., Ltd. (Chongqing, China). Hydrogen peroxide solution (H_2O_2), N, N'-(dithiodi-2,1-ethanediyl) bis(acrylamide) (BACy), and 2,2-Diphenyl-1-picrylhydrazyl (DPPH) were purchased from Macklin Biochemical Technology Co., Ltd. (Shanghai, China). Milli-Q (18.3 MΩ) water was used in all experiments. All chemicals were used as received without further purification. Mouse embryonic fibroblasts (NIH-3T3) were cultured in Dulbecco's Modified Eagle Medium (DMEM) supplemented with 10% Newborn Calf Serum (NCS) and 1% penicillin-streptomycin (P/S) solution. These reagents were procured from Punosai Life Sciences Co., Ltd. (Wuhan, China).

Synthesis of ROS-responsive crosslinkers

For the synthesis of N, N'-(diselanediylbis (ethane-2,1-diyl)) diacrylamide (SOA crosslinker), 500 mg of SD was dissolved in a solvent mixture consisting of 1 mL of water and 5 mL of DMF. DIPEA (6.0 eq.), AAc (3.0 eq.), and PyBOP (3.0 eq.) were added sequentially until complete dissolution was achieved. The mixture was stirred at room temperature for 30 min. Subsequently, the solvents were removed under reduced pressure via rotary evaporation. The crude product was purified using column chromatography on silica gel, eluting with a DCM: MeOH mixture (20:1 v/v). The solvent from the eluate was then evaporated under reduced pressure to yield the SOA product. The desired product was yielded with a recovery rate of approximately 90%.

To synthesize N,N'-((propane-2,2-diylbis(sulfanediyl)) bis(ethane-2,1-diyl)) diacrylamide (TKA crosslinker), 200 mg of TK-NH_2 was dissolved in 2 mL of DCM. DIPEA (3 eq.) was then added, followed by the controlled addition of acryloyl chloride (2 eq.) under an ice bath. The reaction proceeded at room temperature for 30 min. Subsequently, the reaction mixture was gathered under reduced pressure and purified using column chromatography with a DCM: MeOH mixture (10:1 v/v). The desired product was yielded with a recovery rate of approximately 78%.

Synthesis of the degradable Hydrogels

PAAm hydrogels were prepared by dispersing 4M AAm in 5 mL DI water, adding 200 μL LAP solution (2.5% (w/w)) and SOA crosslinker (0.4 g/ml DMSO) to achieve C values of 2.1×10^{-3}, 4.2×10^{-3}, and 8.4×10^{-3} (C is the crosslinker-to-monomer molar ratio). The mixture was ultrasonically dispersed for 5 minutes. For thermosetting PAAm hydrogels, UV initiator was replaced by ACVA. For PNIPAm and PAAc hydrogels, 4M AAm was replaced by 4M NIPAm and 30 wt% AAc, respectively.

To fabricate SeDH, 15 wt% AAc and 1 wt% AAc-NHS were mixed, followed by the addition of 200 μL 2.5% LAP and 12 mg SOA. The mixture underwent UV curing (365 nm, UVP CL-100) for 8 min. This hydrogel was then immersed in a solution containing 30 wt% AAc, 1 wt% AAc-NHS, 48 mg SOA, and 3 mL of 2.5% LAP (100 mL total) for 24 hours to reach swelling equilibrium, followed by another 8 minutes of UV curing. To ensure that the hydrogel was stretched only in the thickness direction, the hydrogel was dried under biaxial stretching ($\lambda = 2$). The final SeDH was dehydrated to produce a film of 250 ± 50 μm thickness for adhesion testing. The hydrogels prepared by substituting the SOA crosslinker with equivalent molar amounts of TKA and MBAA are referred to as TKDH and MBDH, respectively.

For DLP 3D printing defect tissue filling hydrogels, 30 wt% AAc and 1 wt% AAc-NHS were mixed, followed by the addition of 1 mL 2.5% LAP and 24 mg SOA (every 5 mL). When printing, the pattern drawn through SolidWorks software is converted into G-code format, with each layer having a printing time of 5 s.

In terms of hydrogels with adjustable degradation times, SOA in PAAm hydrogels was replaced with an equivalent molar mixture of SOA and TKA ($C_{SOA+TKA}=4.2\times10^{-3}$), with ratios adjusted to 1:0, 4:1, 2:1, 1:1, 1:2, and 0:1 to explore the impact on mechanical properties and degradation behavior.

Characterizations

Electrospray Ionization (ESI) mass spectra were recorded by matrix-assisted laser desorption ionization time of flight mass spectrometry (MALDI-TOF MS) (Bruker ultraflextreme, Germany). The ^{1}H NMR spectra of the samples were recorded on a 400 MHz Bruker NMR spectrometer (Bruker 400 MHz, Germany). Fourier transform infrared (FT-IR) spectroscopy (Nicolet iS20, US) was characterized by the tablet pressing method using a scanning range from 4,000 to 800 cm^{-1} at a resolution of 4 cm^{-1}. X-ray photoelectron spectroscopy (XPS) (Thermo Scientific K-Alpha, US) measurements were performed with Si wafers as the substrates. Gel permeation chromatography (GPC) (Agilent GPC 50) was used for polymeric molecular weight determination (eluent: water). The capability of SOA to scavenge DPPH radicals was tested by a UV-Vis spectrophotometer (DR6000, US) to explore its anti-oxidation property. The SOA (4 mg) was mixed quickly with 200 μM DPPH ethanol solution (2.5 mL) in the dark. Then the absorbances of the mixture (DPPH/SOA) solution (A_{sample}) and DPPH ethanol solution ($A_{control}$) were detected at 517 nm for several times within 115 min. The ability of clearing DPPH radicals was calculated according to $(A_{control}-A_{sample})/A_{control}\times100\%$. The hydrogels were frozen in liquid nitrogen,

followed by freeze-drying for three days to prepare for microstructural characterization. The microstructure and elemental composition of the samples, after sputtering a thin layer of gold, were observed using a benchtop scanning electron microscope (SEM 3500, Japan).

Mechanical tests

The mechanical properties of the hydrogels were tested by a universal testing machine (Instron 5965, USA) with a 10 N or 500 N load cell at a speed of 100 mm/min at room temperature.

To measure tensile strength, dumbbell-shaped specimens (length 20 mm, width 4 mm, thickness 2 mm) were used. Tensile strain was defined as the change in length divided by the original length. The Young's modulus was calculated from the slope of the nominal stress-strain curve within the 0-5% range. The same method was employed to test the stress-strain behavior and modulus during degradation, with dimensions measured 6 times using a caliper to obtain an average value. For dry film tensile tests, the dimensions of the films were 20 mm in length, 2 mm in width, and 0.25 mm in thickness. For swollen SeDH, fracture toughness tests were conducted using pure shear specimens in tension, with specimen dimensions of 50 mm in length, 2 mm in thickness, and 10 mm in height, and a crack size of 20 mm. Fracture toughness was determined based on tensile tests using both notched and unnotched specimens.

To investigate the effects of degradation and swelling on adhesion performance, topological adhesion was formed using polyester fabric as the adherend. The prepared hydrogel samples were dried and then soaked in either 1% (w/w) H_2O_2 or pure water for 3 min as the initial testing condition. The samples were stored in a humid environment at 37℃ (either in 1% (w/w) H_2O_2 or pure water), and adhesion performance was tested at different time intervals. During testing, PET films (thickness 50 μm) were used as the rigid backing. The test sample width was 10 mm, and a pre-cut of approximately 10 mm in length was made. A 180° peel test was conducted, and the energy release rate was calculated as 2F/w, where F is the measured force and w is the width of the test sample.

To measure the interfacial toughness of SeDH, adhesive samples with a width of 1.5 cm were prepared and subjected to a standard 180-degree peel test. When the peeling process stabilized, the force measured reached a steady state. Interfacial toughness was determined by calculating twice the plateau force divided by the width of the sample. PET films (thickness 50 μm) were used as the rigid backing for tissues and engineered solids, bonded with cyanoacrylate adhesive.

To measure the shear strength of SeDH, adhesive samples with dimensions of 1.5 cm in width and 1.5 cm in length were prepared for a standard lap shear test. The shear strength was determined by dividing the maximum force by the adhesion area.

Preparation of engineering solids

Ecoflex, PDMS, and PI films were cleaned with ethanol, followed by treatment with oxygen plasma for 2 min. Subsequently, the films were immersed in a 1 vol/vol%

aqueous solution of APTES for 3 hours at room temperature. Before use, the substrates were rinsed with ethanol and dried with nitrogen flow. For the hydrogels, a solution containing 50 μL of 0.1 M MBAA and 200 μL of 2.5% (w/w) LAP was prepared, into which 4M AAm monomer was added. The mixture was then photocured for 5 min.

Preparation of hydrogel coatings on PVC catheters

The hydrogel precursor solutions were prepared by dispersing 4 M AAm in 5 mL of PVA solution, adding 200 μL of LAP solution (2.5% (w/w)) and SOA crosslinker to achieve C_2. A 2% water-soluble red dye was added to the precursor solution for easy visualization. The PVC catheters were first cleaned with isopropanol, then dried under a nitrogen stream. The substrates were treated with atmospheric plasma using a plasma cleaner for 3 minutes. The catheters treated with plasma were then dipped in an acetone solution containing benzophenone (BP) initiator for 3 minutes and dried again under a nitrogen stream. The catheters were immersed in the hydrogel precursor solution and then slowly withdrawn, utilizing the viscosity of PVA to form a gel layer on the surface. The catheters were then exposed to 365 nm UV light for 1 minute, repeating the process 3-5 times. Through the action of both BP and LAP initiators, the PAAm/PVA hydrogel was cured and covalently bonded to the surface of the PVC tubes.

Research on drug release in vitro

Hydrogels with a crosslinker to monomer molar ratio of C_2 were prepared and immersed in a 1 mg/mL tetracycline hydrochloride aqueous solution until swelling equilibrium was achieved. The drug-loaded hydrogels were then placed in H_2O_2 solution and shielded from light at 37℃ for drug release. Every so often, 5 mL of the solution was withdrawn for absorbance measurement using a UV-visible spectrophotometer. Afterwards, 5 mL of aqueous solution was added back into the original solution, and the concentration was calculated using the standard curve of tetracycline hydrochloride. The cumulative drug release rate was calculated using the following formula (1):

$$D = \frac{\sum_{i=1}^{n-1} C_i V_i + C_n V}{W} \tag{1}$$

where C_i (i=1,…,n-1) and C_n represent the concentrations of tetracycline hydrochloride in the solution at that moment. V_i denotes the volume of the solution withdrawn at the i-th time, which is 5 mL for all i. V signifies the total volume of the solution, namely 50 mL. The denominator W gives the amount of tetracycline hydrochloride loaded in the hydrogel, measured in mg.

In vitro biodegradation tests

We conducted in vitro biodegradation experiments on ROS-responsive hydrogels using varying concentrations of H_2O_2. To facilitate visualization, a small amount of PEDOT:PSS was added to the AAm hydrogel precursor solution prior to photocuring. Colorless hydrogels were subjected to macroscopic degradation observations using 1%

(w/w), 0.1% (w/w), and 0.01% (w/w) H_2O_2. Similar methodologies were applied to characterize the macroscopic degradation of other hydrogels. The hydrogels were immersed in different concentrations of H_2O_2 and pure water for designated durations to compare mechanical properties through tensile testing. Dry hydrogels with a diameter of 5 mm were immersed in different concentrations of H_2O_2 to evaluate swelling and mass loss. Controllable degradation hydrogels were tested for mechanical properties, swelling, and mass loss in 0.1% (w/w) H_2O_2 (25℃ or 37℃) using a similar protocol. Swelling and mass loss were calculated according to the following formulas (2,3):

$$\text{Relative remaining mass} = W_t/W_0 \quad (2)$$

$$\text{Relative mass} = \frac{W_{Dt}}{W_0} \times 100\% \quad (3)$$

where W_0 represents the initial weight of the hydrogel in a dry state, W_t denotes the weight of the hydrogel after water absorption at various time instants, and W_{Dt} indicates the weight of the hydrogel after freeze-drying at different time instants. For each data point, three parallel samples were carried out, and the data are presented as mean S.D. (n = 3).

To simulate the degradation of SeDH under low-concentration conditions, fully swollen gels were immersed in 100 mL of PBS solution for 9 hours. Subsequently, 10 μL of 1% (w/w) H_2O_2 solution was added. The mass of the hydrogel was measured at intervals to evaluate the degradation of SeDH in vivo environment.

In vitro biocompatibility tests

We utilized SeDH hydrogels to conduct in vitro biocompatibility tests for cell culture. To prepare the conditioned medium for these assays, we incubated batches of 20 mg hydrogels (SeDH, TKDH, and MBDH, respectively) in 1 mL of DMEM at 37℃ for 24 hours. Unmodified DMEM served as the control. To assess cell viability quantitatively, NIH-3T3 cells were seeded in a 96-well plate (10^3 cells/well) and treated with the SeDH-/TKDH-/MBDH-conditioned medium. The cells were incubated at 37℃ in a 5% CO_2 environment for 1, 3, and 5 days. Cell viability was quantified using a CCK-8 assay kit by adding 10 μL of CCK-8 solution to each well and incubating for an additional 2 hours at 37℃. Absorbance was measured at 450 nm using a multimode plate reader (BIO-RAD iMARK, Japan). Cells treated with unmodified DMEM were used as the reference, with their viability defined as 100%. For each data point, eight parallel experiments were conducted, and the results are presented as the mean ± SD (n = 8).

Similarly, NIH-3T3 cells were used to evaluate the cytotoxicity of the hydrogels. NIH-3T3 cells were seeded in a 96-well plate (10^3 cells/well) and then treated with SeDH-/TKDH-/MBDH-conditioned medium. The cells were incubated at 37℃ in a 5% CO_2 atmosphere for 1, 3, and 5 days. Cell cytotoxicity was qualitatively assessed using a Calcein-AM/PI staining kit. Fluorescent images of the cultured cells were captured using an inverted fluorescence microscope (DM i8, Germany) at excitation/emission wavelengths of 490/515 nm and 535/617 nm to differentiate between live (green) and dead (red) cells.

The in vitro biocompatibility of SeDH degradation products was evaluated using the same method.

In vitro hemocompatibility tests

In vitro hemocompatibility evaluations were performed using SeDH hydrogels. Prior to the experiments, blood samples were collected from KM mice (18-22 g, 6-8 weeks, purchased from Beijing Vital River Laboratory Animal Technology Co., Ltd., Suzhou Branch). The experiment was designed with three groups: a negative control group (normal saline), a positive control group (ultrapure water), and a sample group (normal saline containing SeDH hydrogel extract).

I. Preparation of red blood cell (RBC) suspension

Anticoagulated whole blood was centrifuged at 1000 rpm for 10 min. Then, 0.2 mL of the lower red blood cell precipitate was pipetted into a 1.5 mL centrifuge tube, followed by the addition of 1.0 mL of normal saline. The centrifuge tube was gently inverted to mix the contents uniformly, and centrifugation was repeated at 1000 rpm for 10 min. After carefully aspirating the supernatant from the centrifuge tube, 1.4 mL of normal saline was added to the tube to dilute the red blood cells. Three tubes of red blood cell suspension were prepared using the same method and set aside for subsequent use.

II. Hemolysis test procedure

A 200 μL volume of RBC suspension was added to each test tube of the experimental groups, followed by the addition of the corresponding solution (SeDH hydrogel extract for the sample group; the respective solvent for the control groups). After thorough mixing, the mixtures were incubated at 37 ℃ for 24 h. Post-incubation, the mixtures in the test tubes were centrifuged at 800 rpm for 15 min. The supernatants were collected and transferred to a 96-well microplate. The 96-well microplate was placed in a microplate reader, with the detection wavelength set at 545 nm to determine the absorbance value of each well. The experiment was conducted in triplicate, and the absorbance data for each group were recorded.

The formula for calculating the relative hemolysis rate of RBCs is as follows:

$$\text{Relative hemolysis rate } (\%) = \frac{D_t - D_{nc}}{D_{pc} - D_{nc}} \times 100\% \quad (4)$$

where *D*□ represents the absorbance of the experimental sample, *D*□*c* represents the absorbance of the negative control group, and *D*□□ represents the absorbance of the positive control group. According to the evaluation criteria for hemolytic performance of medical materials: when the red blood cell (RBC) hemolysis rate is ≤ 5%, it indicates that the material meets the hemolysis rate requirements for medical materials and will not induce severe hemolytic reactions. When the RBC hemolysis rate is > 5%, it suggests that the material may trigger hemolytic reactions.

In vitro hemostasis test of simulated blood circulation system

In order to verify that the adhesive strength of the adhesive can withstand the higher blood flow or mechanical stress of organisms with higher blood pressure, the

simulated blood vessels were installed at the water outlet and inlet of a blood circulation pump (with a blood vessel inner diameter of 4 mm, a maximum blood flow rate of 1500 mm/min, and a maximum blood pressure of 28 kPa). The other end of the simulated blood vessel was connected to a section of about 10 cm long arterial blood vessel of a New Zealand white rabbit, and a 2-mm wound was made in the middle position. Then, SeDH was directly applied to the wound site, the flow rate was adjusted to almost the maximum, and the sealing effect was recorded.

Ex vivo adhesion test

To assess the sealing of damaged cardiac tissue, a wound approximately 20 mm in length was created on a porcine heart. To seal the wound, SeDH was applied directly to the incision (either as a single-sided or double-sided adhesive), and pressure was applied for 5 seconds. The sealed heart was then maintained at room temperature for 6 hours to monitor the efficacy of the SeDH-based closure.

For the evaluation of sealing in damaged liver tissue, a wound approximately 30 mm in length was incised on a porcine liver. SeDH was applied to the wound area (either as a single-sided or double-sided adhesive), and pressure was applied for 5 seconds. The sealed liver was subsequently stored at room temperature for 6 hours to assess the closure provided by SeDH.

To evaluate the use of SeDH powder for sealing irregular wounds, an irregular incision was made on a porcine liver. The powder was sprinkled onto the wound area and compressed for 10 seconds to evaluate the seal. The adhesion toughness of the powder was tested by applying it to the porcine liver.

In vivo adhesion, biodegradation, and biocompatibility tests

All animal handling procedures were conducted in accordance with the ethical guidelines approved by the Ethics Committees of Zhejiang University and Fudan University, and all surgical interventions were authorized by the Animal Care Committees (Protocol Number: 2024188 and 2023-09-YJ-QJP-82). In vivo adhesion strength testing on the dynamic heart and hemostasis testing on the liver utilized male Sprague Dawley (SD) rats (200-300 g, 7-8 weeks, sourced from Hangzhou Hulk Biotech Co., Ltd.) and New Zealand rabbits (2.6 kg, 30 weeks, purchased from Hangzhou Medical College). For in vivo gel degradation and histological analysis, C57BL/6J mice were used (weight and supplier unspecified). Prior to experimentation, dry hydrogels were cut into circular thin slices using a sterile 5- or 10-mm punch and were sterilized under UV light for 30 minutes before in vivo application.

a. Dynamic cardiac adhesion test

The dynamic adhesion properties of SeDH bioadhesive were assessed through implantation on the hearts of SD rats. The rats were anesthetized with a 1% pentobarbital anesthesia solution. A thoracotomy was performed at the third or fourth left intercostal space to expose the heart. The SeDH patch was placed on the epicardial surface of the heart. To achieve adhesion, the patch was gently pressed with tweezers for 5 seconds. Adhesion was confirmed by observing the movement of the entire heart while gently pulling at the edges of the patch. The same experiment was

performed in rabbits.

b. In vivo hepatic hemostasis

The hemostatic efficacy of SeDH was evaluated using a liver perforation model in SD rats. The untreated bleeding group served as the control. Rats were anesthetized, and the liver was externalized onto the surface of a filter paper. A 3 mm bleeding lesion was created using a circular punch, and SeDH was directly applied to the bleeding site. The hemostasis process was documented using a camera. Blood loss was quantified by measuring the total weight of blood absorbed by the filter paper. The same experiment was performed in rabbits.

c. In vivo hydrogel degradation

SeDH, TKDH, and MBDH were shaped into 5 mm diameter discs using a circular punch. Before implantation, the patches were sterilized under UV light. Mice were anesthetized, and a 1-2 cm incision was made on the lateral back to implant the SeDH, TKDH, and MBDH, with no more than three patches implanted per mouse. The incision was subsequently sutured closed. At designated post-implantation intervals (1, 2, and 3 weeks), three mice per type of hydrogel were sacrificed to collect the implanted SeDH, TKDH, and MBDH samples. These samples were thoroughly washed with deionized water, then dried and weighed. The change in mass was used to quantify and compare the in vivo biodegradation rates of the different crosslinkers.

d. Histological analysis

To evaluate the in vivo biocompatibility of the hydrogel samples (SeDH, TKDH, and MBDH), subcutaneous tissues that were in close contact with the hydrogels were sampled at the time of the final collection. The tissues were fixed in a 4 vol.% solution of paraformaldehyde, embedded in paraffin. These samples were sectioned and subjected to histological analysis using hematoxylin and eosin (H&E) staining.

e. In vivo metabolic pathways

To characterize the metabolic pathway of adhesive degradation products in vivo, hydrophilic CY7 with fluorescent properties (excitation wavelength: 740-750 nm, emission wavelength: 770-780 nm) was used to modify polyacrylic acid (PAA, M_w~100 kDa, degradation products of hydrogel) chains that were nearly identical in structure to the degraded macromolecular chains (with an average of 5 CY7 molecules grafted per chain), yielding PAA-CY7 conjugates. These conjugates were formulated into a 5 mg/mL solution in normal saline. Male Balb/c-nu mice (20-22 g, 6-8 weeks, purchased from Beijing SPF Biotechnology Co., Ltd.) were selected. Each mouse was injected with 200 μL of PAA-CY7 solution via abdominal subcutaneous injection or h intraperitoneal (hepatic surface) injection to simulate the degradation process of the adhesive after application on subcutaneous tissues or surfaces of organs such as the liver. A multimodal in vivo animal imaging system was used to monitor the metabolic pathway of PAA-CY7 at different time points in vivo. Finally, the major organs (heart, liver, spleen, lung, kidney) and blood of mice were collected 6 days after administration for fluorescence intensity detection to verify the final destination of the macromolecular chains.

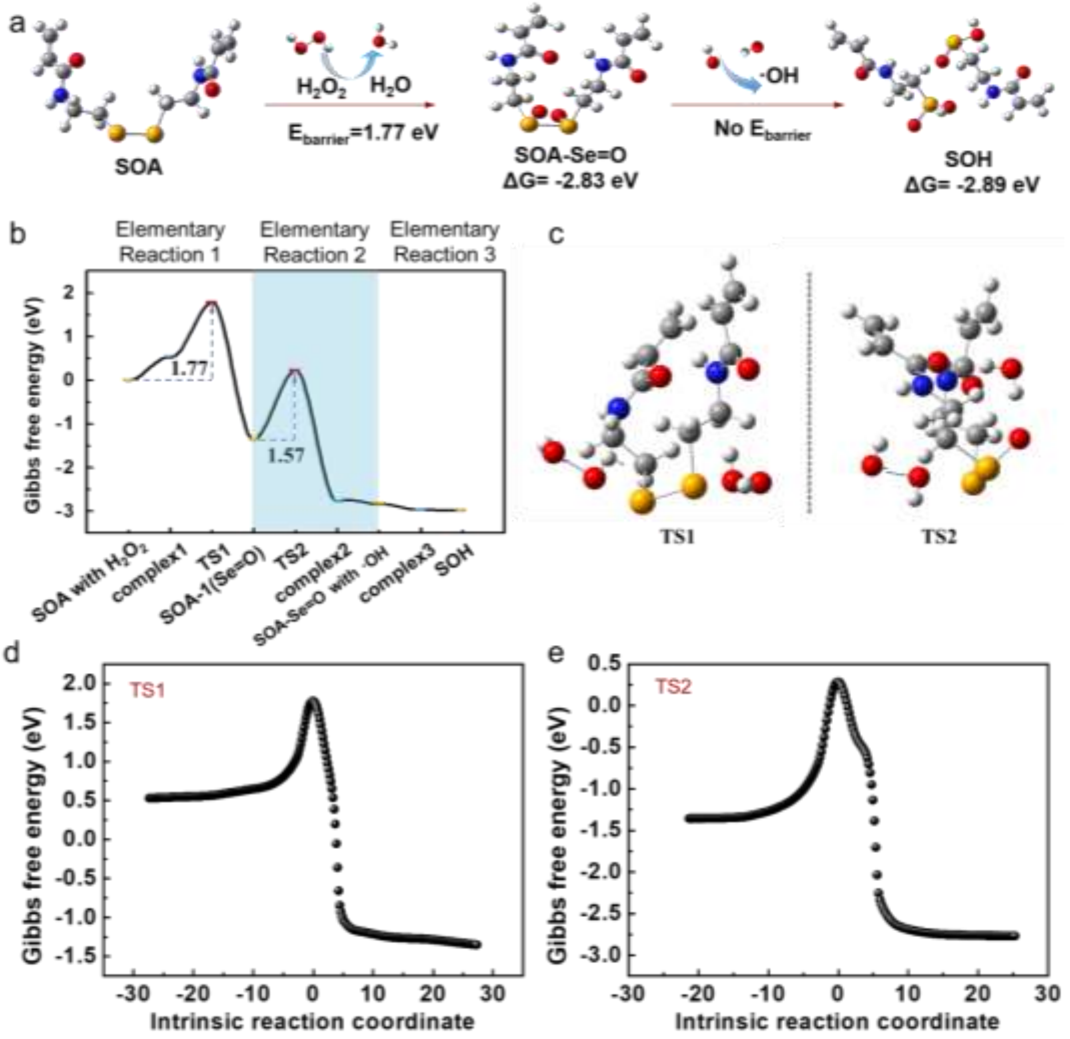

Fig. S1. Calculation of the degradation process of low-redox-stress-threshold SOA crosslinker. (a) Calculated degradation pathway of SOA crosslinker in response to H_2O_2 as a stimulus. (b) Thermodynamic processes corresponding to the degradation pathway of the SOA crosslinker. (c) Structures TS1 and TS2. (d and e) The intrinsic reaction coordinate (IRC) of the transition state TS1 (d) and TS2 (e). The IRC result shows that a smooth connection from the transition states to both reactant and product sides, indicating that the obtained transition state is highly reliable.

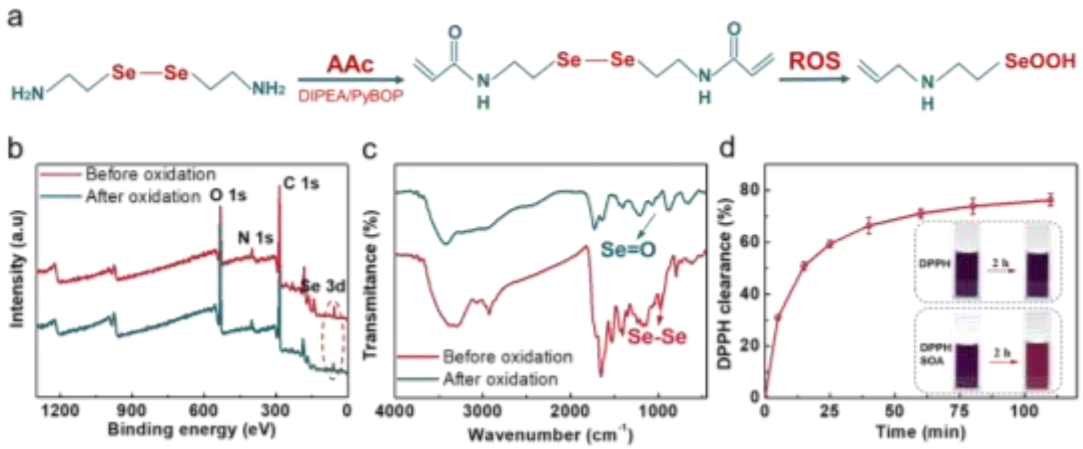

Fig. S2. Synthesis route, structural characterization before and after oxidation, and antioxidant capacity of SOA crosslinker. (a) Structure of synthesis and degradation products of the SOA crosslinker. (b) XPS spectra of the SOA crosslinker before and after oxidation. (c) FTIR spectra of the SOA crosslinker before and after oxidation. (d) Clearance of DPPH by SOA over time (25℃) (Insets: The macroscopic view of DPPH or DPPH/SOA solution after 2 hours). Values represent the mean and standard deviation (n=3-5). To obtain the designed SOA crosslinker, acrylic acid and selenocystamine dihydrochloride (SD) were subjected to precise chemical synthesis steps, ultimately yielding a hydrophilic SOA crosslinker.

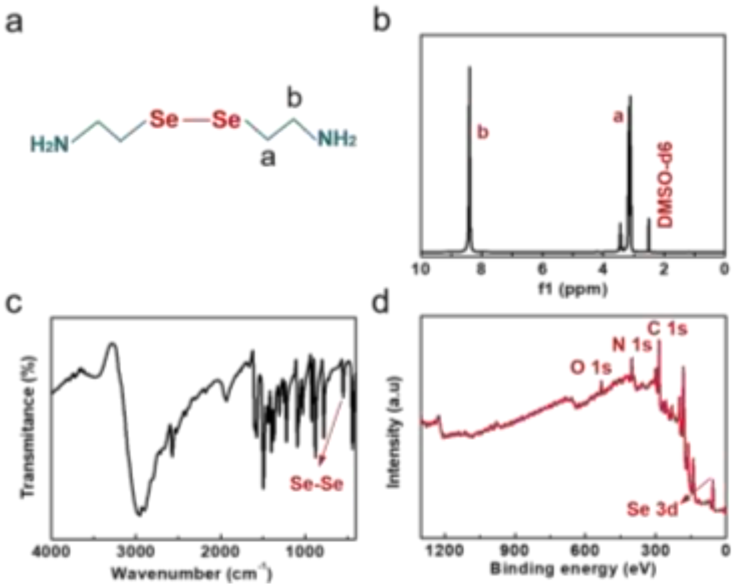

Fig. S3. Structural characterization of selenocystamine dihydrochloride SD. (a-d) The molecular structure (a), ^{1}H NMR (b), FTIR spectrum (c), and XPS spectrum (d) of SD, the raw material for the synthesis of a SOA crosslinker.

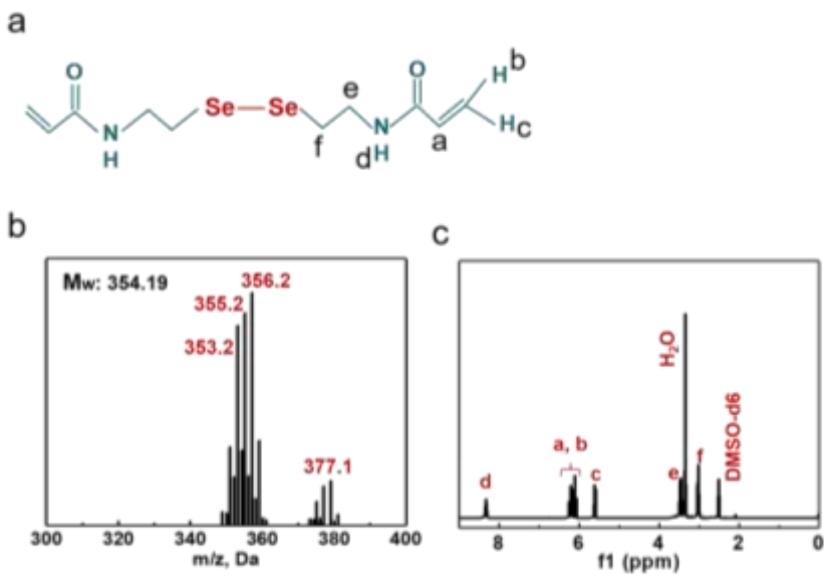

Fig. S4. Structural characterization of SOA crosslinker. (a-c) The molecular structure (a), mass spectrum (b), and ^{1}H NMR (c) of the SOA crosslinker. The accuracy of the SOA synthesis is demonstrated through various characterization techniques, including functional groups and elemental analysis.

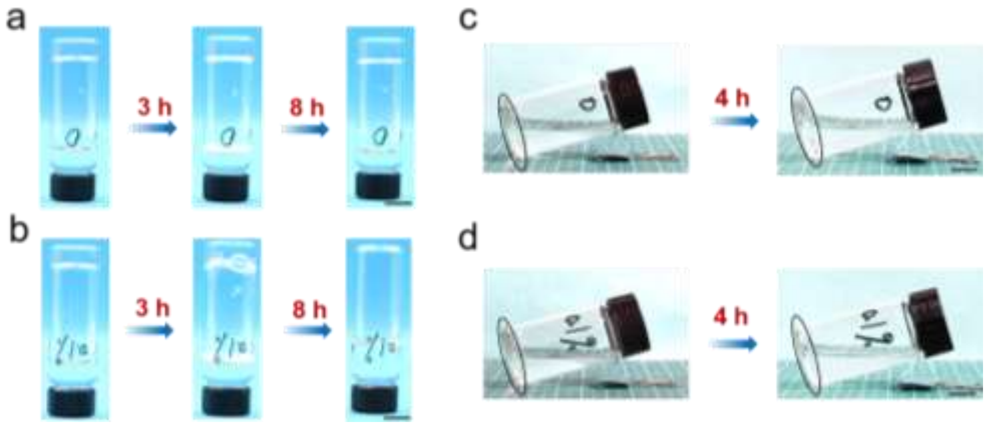

Fig. S5. Schematic diagram of hydrogel degradation with different monomers. (a and b) Comparative degradation diagrams of PNIPAm hydrogels prepared via photo-initiation in pure water (a) and 0.1% (w/w) H_2O_2 (b), respectively (25℃). (c and b) Comparative degradation diagrams of PAAc hydrogels prepared via photo-initiation in pure water (c) and 0.1% (w/w) H_2O_2 (d), respectively (25℃). Similar to PAAm hydrogels, both PNIPAm and PAAc hydrogels swell in pure water. Especially, they exhibit rapid swelling and degradation in low concentrations of H_2O_2. Scale bar: 1 cm.

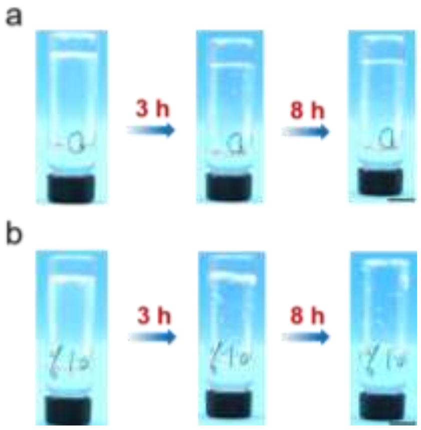

Fig. S6. Comparison of PAAm hydrogels prepared via thermal initiation. (a and b) Comparative degradation diagrams of PAAm hydrogels prepared via thermal initiation in pure water (a) and 0.1% (w/w) H_2O_2 (b) (25℃). Scale bar: 1 cm. In pure water, the hydrogel only experiences swelling, while in 0.1% (w/w) H_2O_2, the crosslinkers in the hydrogel break, eventually causing the macromolecular network to dissolve in the solution.

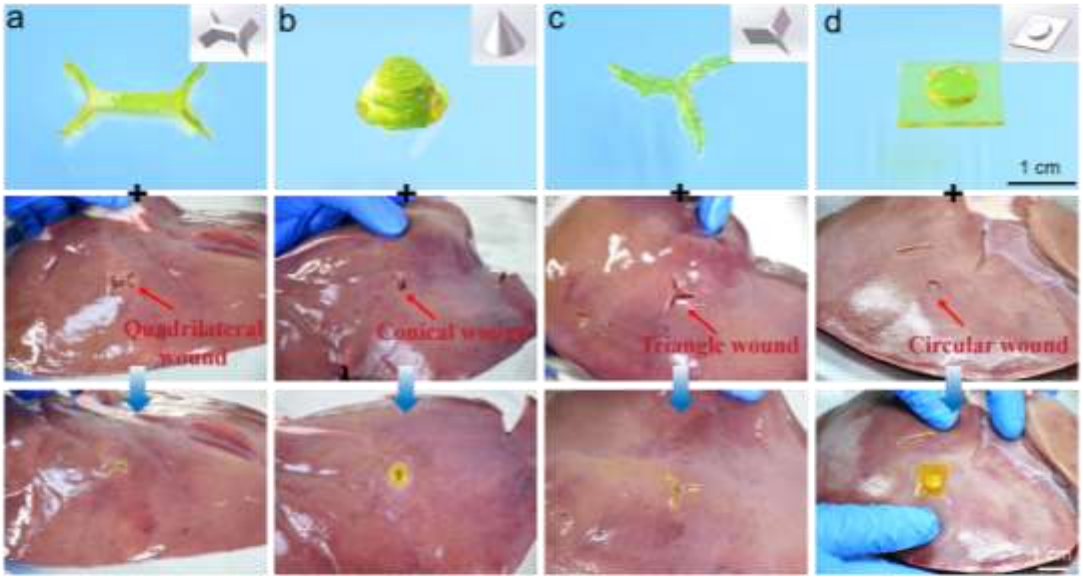

Fig. S7. Diagrams of complex-structured hydrogels with tissue-adhesive properties fabricated via DLP 3D printing to fulfill the needs of tissue defect repair. (a) Quadrangle. (b) Cone. (c) Triangle. (d) Circular.

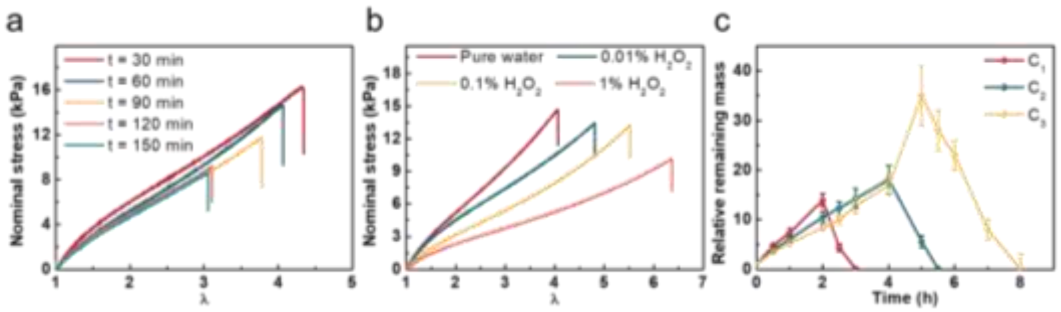

Fig. S8. Mechanical and degradation properties of PAAm hydrogels. (a) Effects of swelling and degradation on the mechanical properties of hydrogels in pure water within 150 min (25 ℃). (b) Mechanical properties of hydrogel (C_2) after reacting for 1 hour in pure water and various concentrations (25 ℃). (c) Change in overall mass of hydrogels with different concentrations of crosslinker in 0.1% (w/w) H_2O_2 over time (25 ℃). Values represent the mean and standard deviation (n = 3-5). When comparing Fig. 2c and Fig. S8a, one observes that the hydrogel rapidly attains swelling equilibrium in pure water, resulting in minimal changes in its mechanical properties. In contrast, the crosslinker within the hydrogel network undergoes continuous degradation in 0.1% (w/w) H_2O_2, and consequently causes a decline in mechanical performance, highlighting the rapid degradation under low-stimulation conditions.

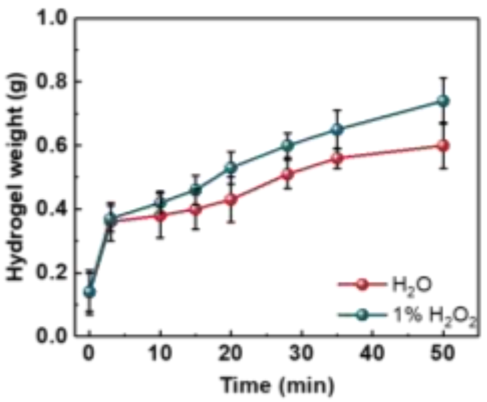

Fig. S9. The variation in hydrogel weight of fast-degrading and non-degrading hydrogels over time in a humid environment (37℃). For tissue adhesives, when the adhesive detaches from the tissue surface, energy is dissipated not only through the rupture of bonds along the crack plane (including covalent and non-covalent interactions) but also through the disruption of numerous sacrificial bonds within the adhesive matrix. It has been demonstrated that the growth of hydrogel chains contributes to the dissipation of a greater amount of energy. This is, when the polymer network is stretched, all C-C bonds between crosslinkers are elastically stretched to their breaking limit before any chain fracture occurs. When a single bond on the chain breaks, the elastic energy stored in the entire chain is released. According to the Lake-Thomas model, the adhesion energy Γ is positively correlated with the square root of the chain length n, i.e., $\Gamma \propto \sqrt{n}$.

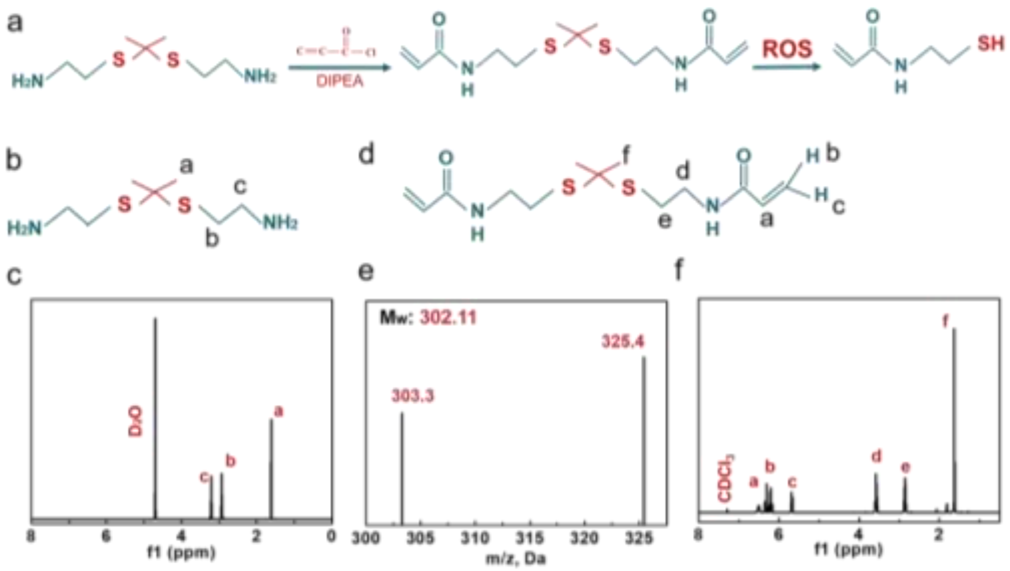

Fig. S10. Synthesis and Degradation of TKA Crosslinker. (a) Chemical structure of the synthesis and degradation of TKA crosslinker. (b) The precursor chemical structure of the TKA crosslinker. (c) ^{1}H NMR of the precursor materials of the TKA crosslinker. (d) Chemical structure of TKA crosslinker. (e and f) Mass spectrum (e) and ^{1}H spectrum (f) of the synthesized TKA crosslinker.

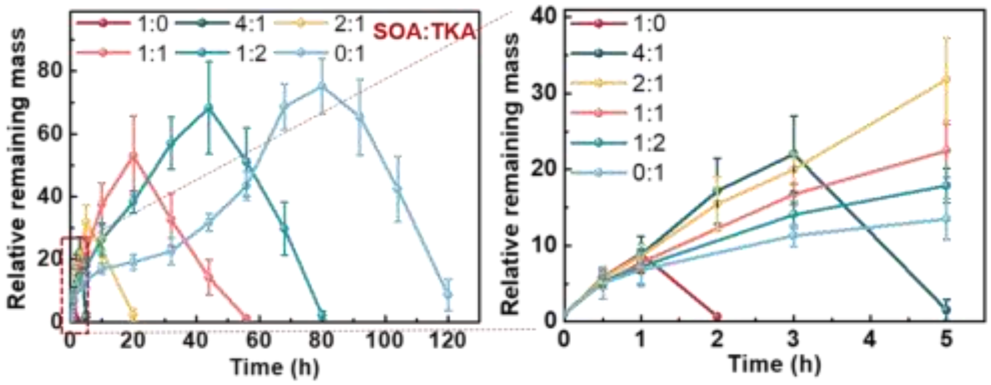

Fig. S11. Relative remaining mass change of SOA/TKA mixed crosslinker hydrogels over time by using 0.1% (w/w) H_2O_2 as stimuli (37℃).

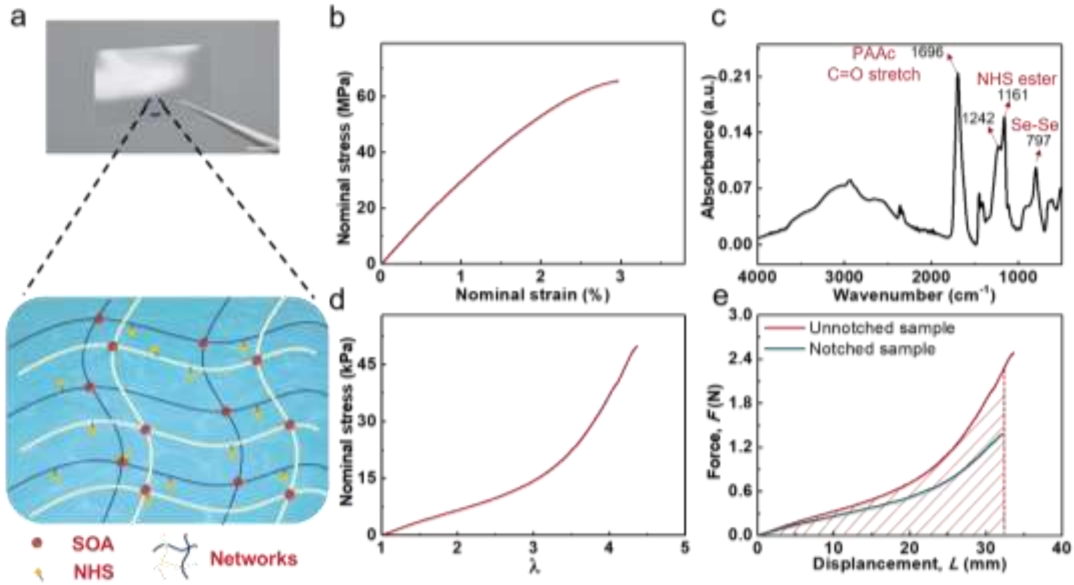

Fig. S12. Performance of SeDH adhesive. (a) Image of the obtained hydrogel dry film, featuring high transparency and adjustable thickness. Scale bar: 1 cm. (b) A nominal stress-nominal strain curve for a dry film. (c) FTIR spectrum of the SeDH with NHS ester. The carboxylic acid C=O stretch at 1696 cm^{-1} is associated with PAAc in the SeDH. The symmetric C-N-C stretch at 1161 cm^{-1} and asymmetric C-N-C stretch at 1242 cm^{-1} are associated with the NHS ester in the SeDH. The peak at 797 cm^{-1} represents the Se-Se bond. (d) A nominal stress versus tensile curve of SeDH in its swollen state, stretched to more than four times its original length. (e) A force versus distance curve between clamps for the unnotched and notched SeDH samples for fracture toughness measurement. To measure the fracture toughness, the method introduced by Rivlin and Thomas is adopted. One sample was unnotched, and the other sample was notched. The unnotched sample was first stretched to record the force-length curve. The fracture toughness (Γ) was calculated by $\Gamma = U(L)/bt$, where b and t represent the width and the thickness of the sample, respectively.

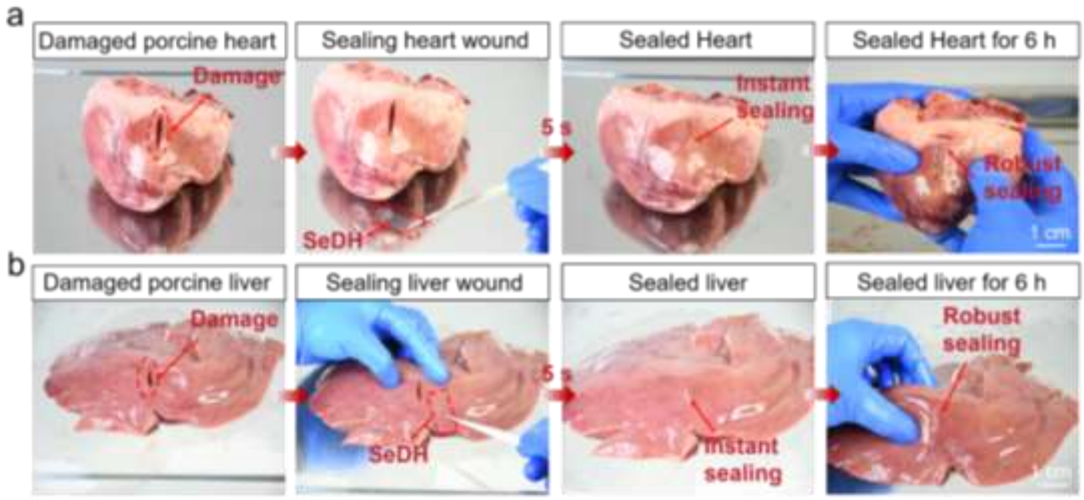

Fig. S13. Dual-sided adhesive applications of SeDH. (a) Sealing of a wounded ex vivo porcine heart by the SeDH. (b) Sealing of a wounded ex vivo porcine liver by the SeDH.

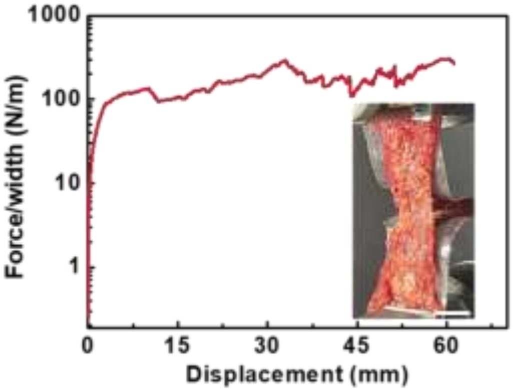

Fig. S14. Adhesive performance of SeDH powder. Force/width versus displacement curves for 180 ° peeling tests of porcine liver adhered by the SeDH powder. Scale bar: 1 cm.

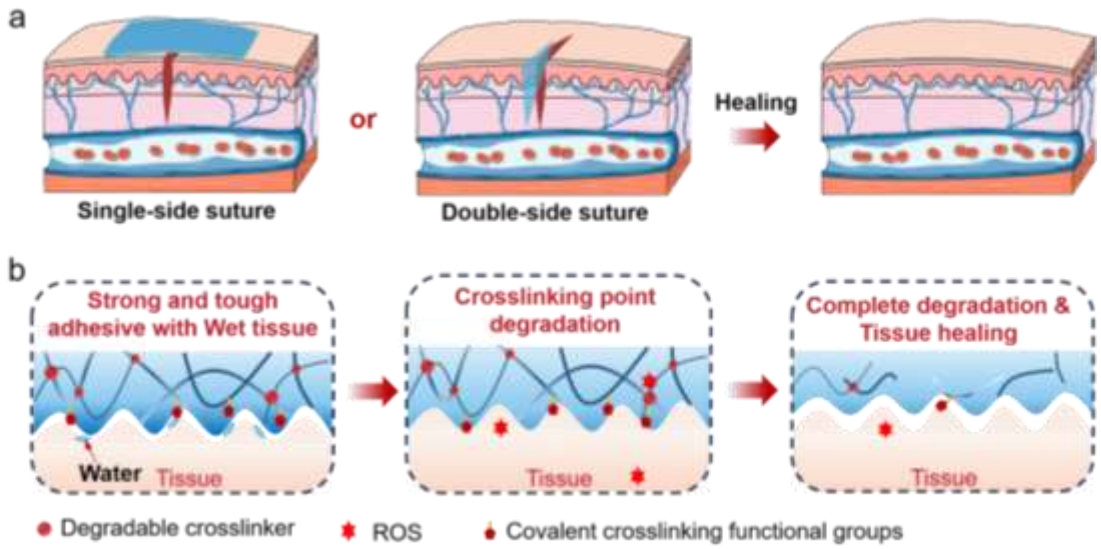

Fig. S15. Schematic illustration of wet tissue adhesion and degradation. (a) applications for tissue adhesion, including single-sided and dual-sided adhesion of SeDH. (b) Degradation of SeDH after adhesion in wet tissue. SeDH can directly adhere to wet tissues (either single-sided or dual-sided) without any additional processes. Upon adhesion to wet tissues, SeDH rapidly swells and absorbs interfacial moisture. Simultaneously, the carboxyl groups on PAAc form temporary bonds with the tissue, followed by the formation of covalent bonds by NHS groups with the tissue. Over time, the production of ROS within the tissue promotes the breaking of crosslinkers within the hydrogel. However, the hydrogel retains its shape until the crosslinking breakage reaches the percolation threshold, at which point the hydrogel fully degrades as the wound heals.

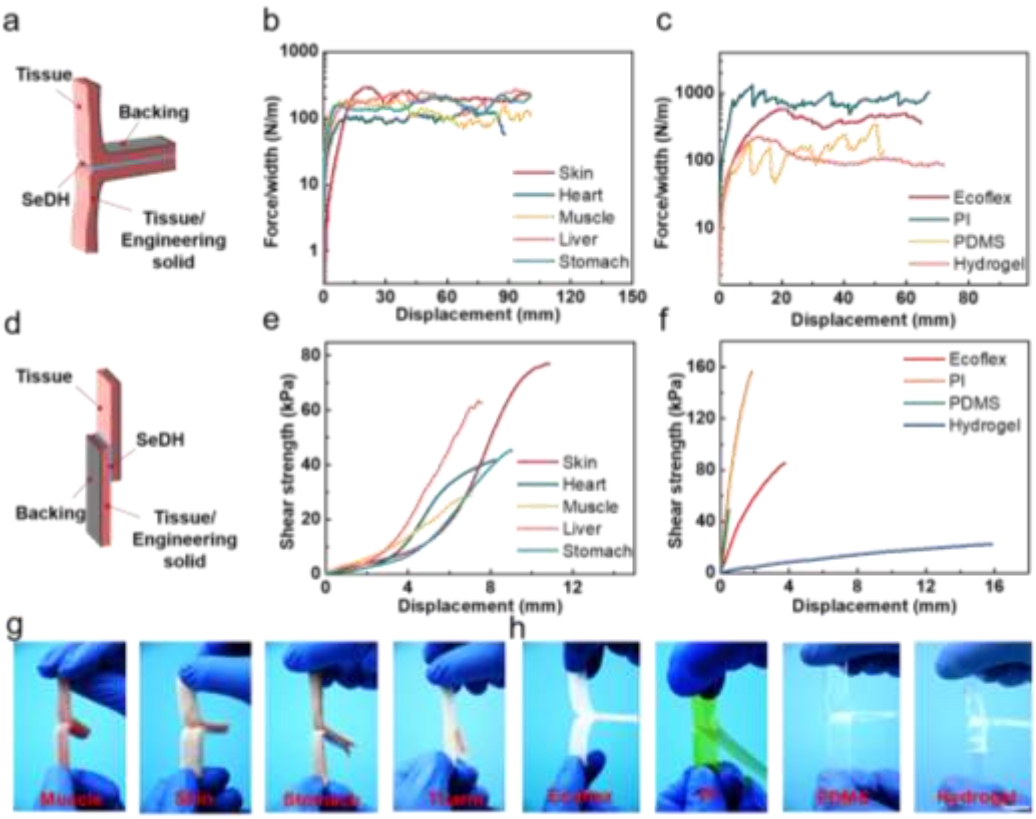

Fig. S16. Mechanical testing of various tissues and engineering solids adhered by SeDH. (a) Schematic of the 180° peel test. (b) Representative peeling curves of various tissues adhered by SeDH. (c) Representative peeling curves of various engineering solids adhered by SeDH. (d) Schematic of the 90° lap-shear test. (e) Representative lap shear test curves of various tissues adhered by SeDH. (f) Representative lap shear test curves of various engineering solids adhered by SeDH. (g and h) Images of adhesion between various ex vivo porcine tissues (g) and engineering solids (h) by SeDH bioadhesive. Scale bar: 1 cm.

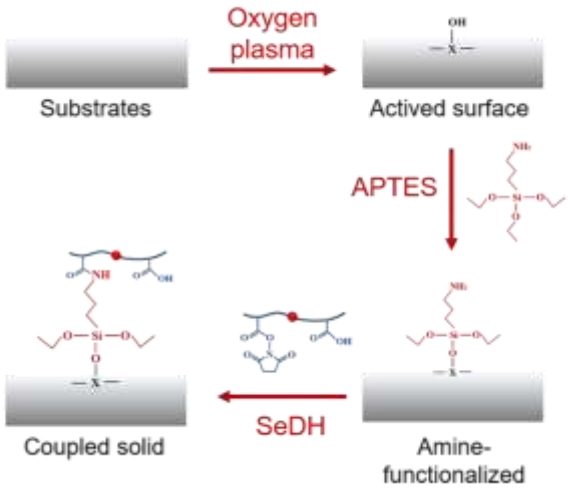

Fig. S17. Schematic of surface functionalization of engineering solids. Schematic of surface functionalization of engineering solids (including Ecoflex, PDMS, and PI), involving surface activation via plasma treatment, followed by immobilization of APTES to generate substrates with primary amine groups on the surface.

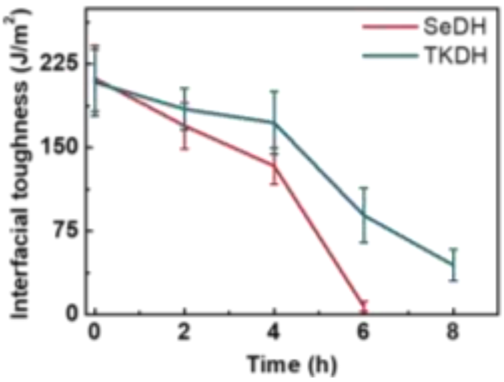

Fig. S18. Time-dependent adhesion performance curves of TKA- or SOA-crosslinked hydrogels bonding porcine skin in 6 h using 0.1% (w/w) H_2O_2 as stimuli (37℃). In the early stage of degradation, the initial adhesion performance of the hydrogels does not decrease rapidly, and this characteristic is conducive to providing necessary mechanical support for the early stage of wound healing.

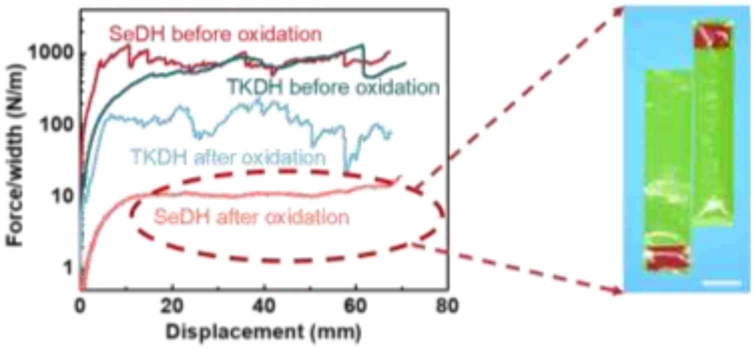

Fig. S19. Adhesion performance of TKA or SOA crosslinked hydrogels bonding PI films for 6 hours using 0.1% (w/w) H_2O_2 as stimuli (37°C). Scale bar: 1 cm.

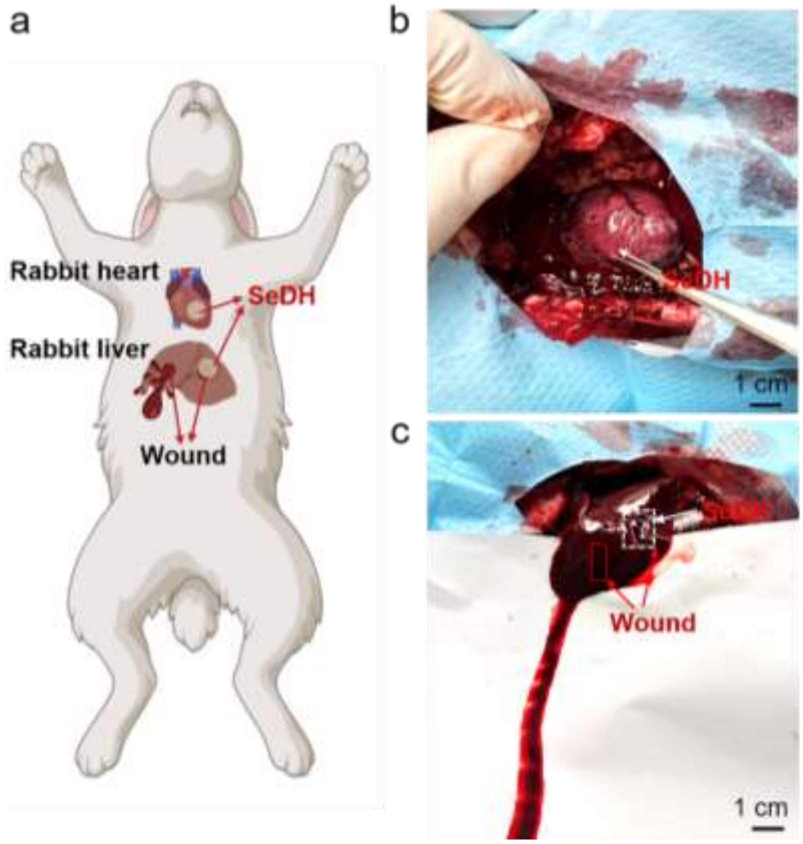

Fig. S20. In vivo adhesion of SeDH in rabbits. (a) Schematic illustration of dynamic heart adhesion and liver wound hemostasis using SeDH in a rabbit model. (b) Adhesion of SeDH on the beating heart of a rabbit in vivo. (c) Adhesion of SeDH on the bleeding liver of a rabbit in vivo.

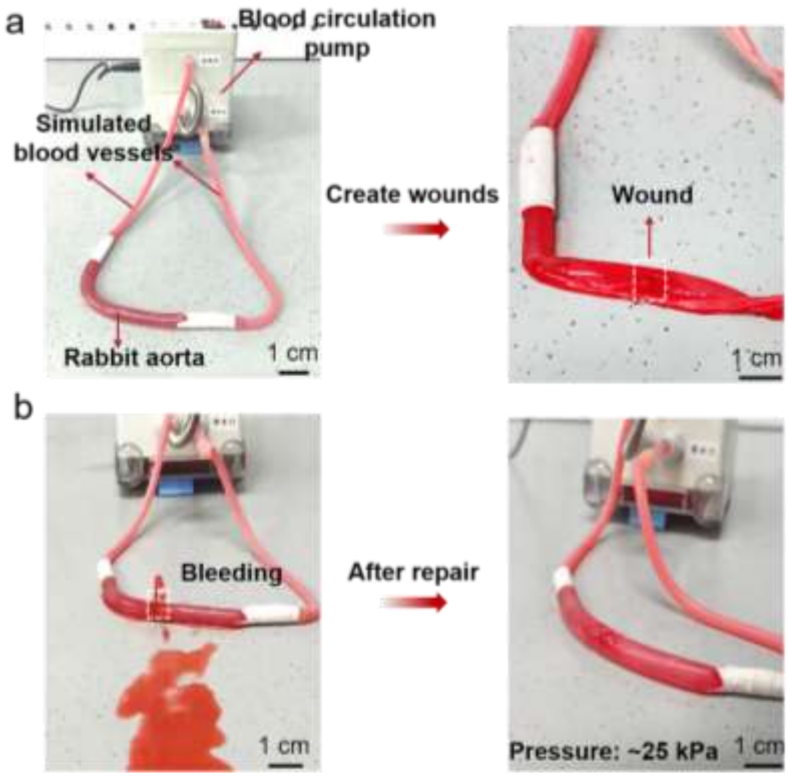

Fig. S21. Hemostatic effect of the in vitro simulated blood circulation system. (a) Schematic diagram of the in vitro simulated blood circulation system and establishment of the vascular wound model. (b) Schematic diagram of the hemostatic effect under hypertensive conditions. The rabbit arterial blood vessels were harvested to construct a simulated blood circulation system, and a wound was made on the blood vessel. When the circulation pump was activated, massive bleeding occurred at the vascular wound. After applying the SeDH adhesive (without additional treatment steps), the bleeding was effectively stopped immediately. Even under simulated hypertension conditions (blood flow pressure ~25 kPa), the adhesive interface remained stable and firm.

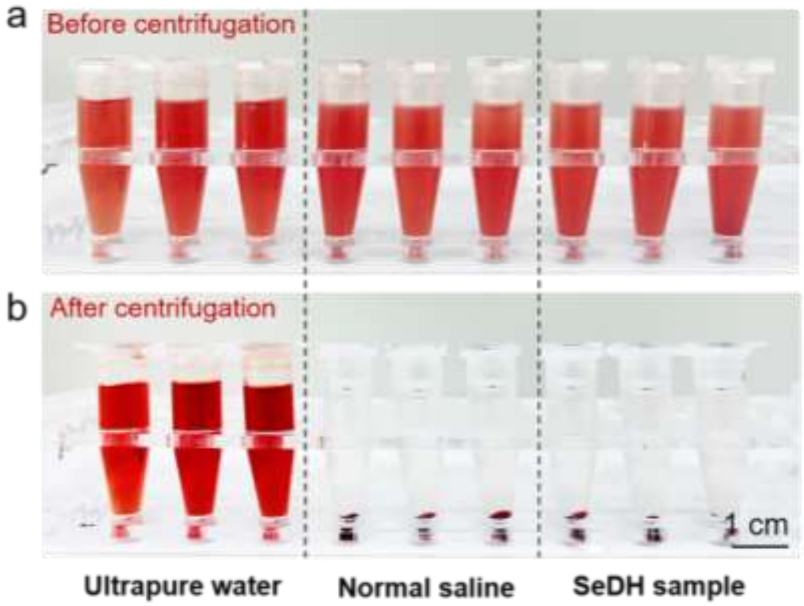

Fig. S22. The hemocompatibility test results of the SeDH. Hemolysis images of the negative control group (normal saline), positive control group (ultrapure water), and sample group (SeDH extract) before centrifugation (a) and after centrifugation (b). The absorbance values of each group measured in the experiment are as follows: the absorbance of the sample group (D□) is 0.050±0.003, the absorbance of the negative control group (D□$_c$) is 0.046±0.002, and the absorbance of the positive control group (D□□) is 1.778±0.022. Substituting these values into Formula (4) for calculation, the hemolysis rate of the sample group is 0.23%. In combination with the qualified standard for medical materials (hemolysis rate ≤ 5%, mentioned earlier), this result indicates that the SeDH hydrogel adhesive possesses excellent blood compatibility.

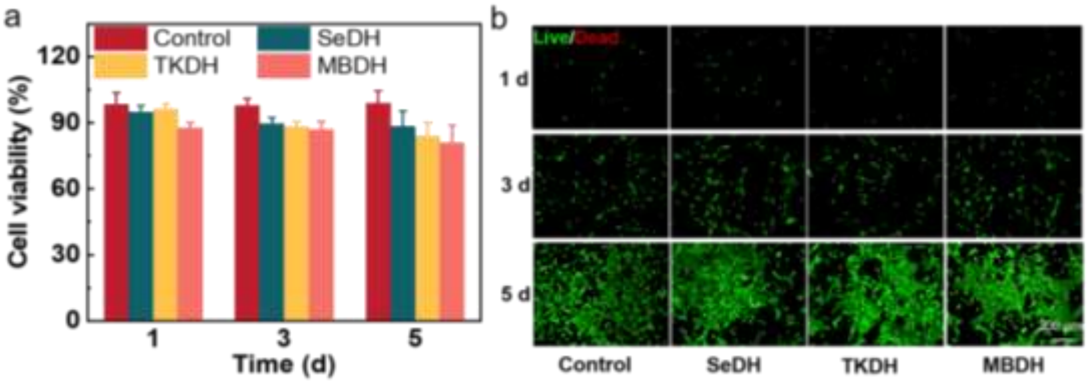

Fig. S23. In vitro biocompatibility tests of different hydrogels (SeDH, TKDH, and MBDH). (a) Cell viability of NIH-3T3 cells cultured with three different conditioned media (SeDH, TKDH, and MBDH) and control media for 1, 3, and 5 days. (b) Representative images of live/dead staining of NIH-3T3 cells cultured with three differently conditioned media (SeDH, TKDH, and MBDH) and control media for 1, 3, and 5 days.

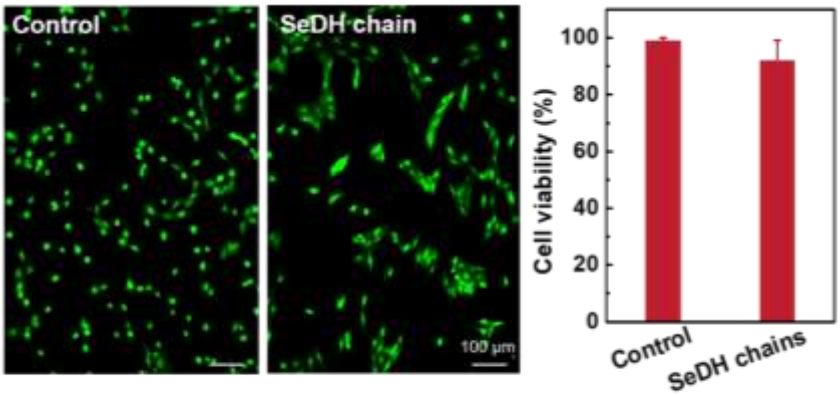

Fig. S24. In vitro biocompatibility testing of SeDH degradation products. In vitro biocompatibility of the molecular chains of SeDH degradation products in a live/dead assay of Mouse embryonic fibroblasts (NIH-3T3) cultured after 24 h.

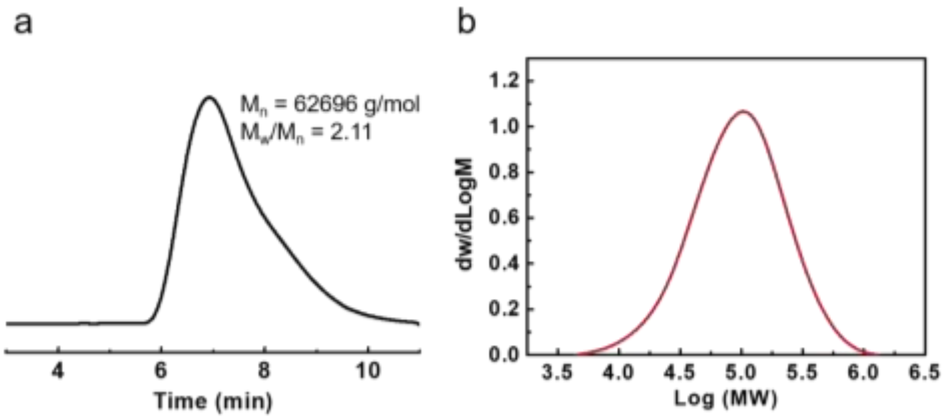

Fig. S25. Gel Permeation Chromatography (GPC) of the molecular chains of SeDH degradation products.

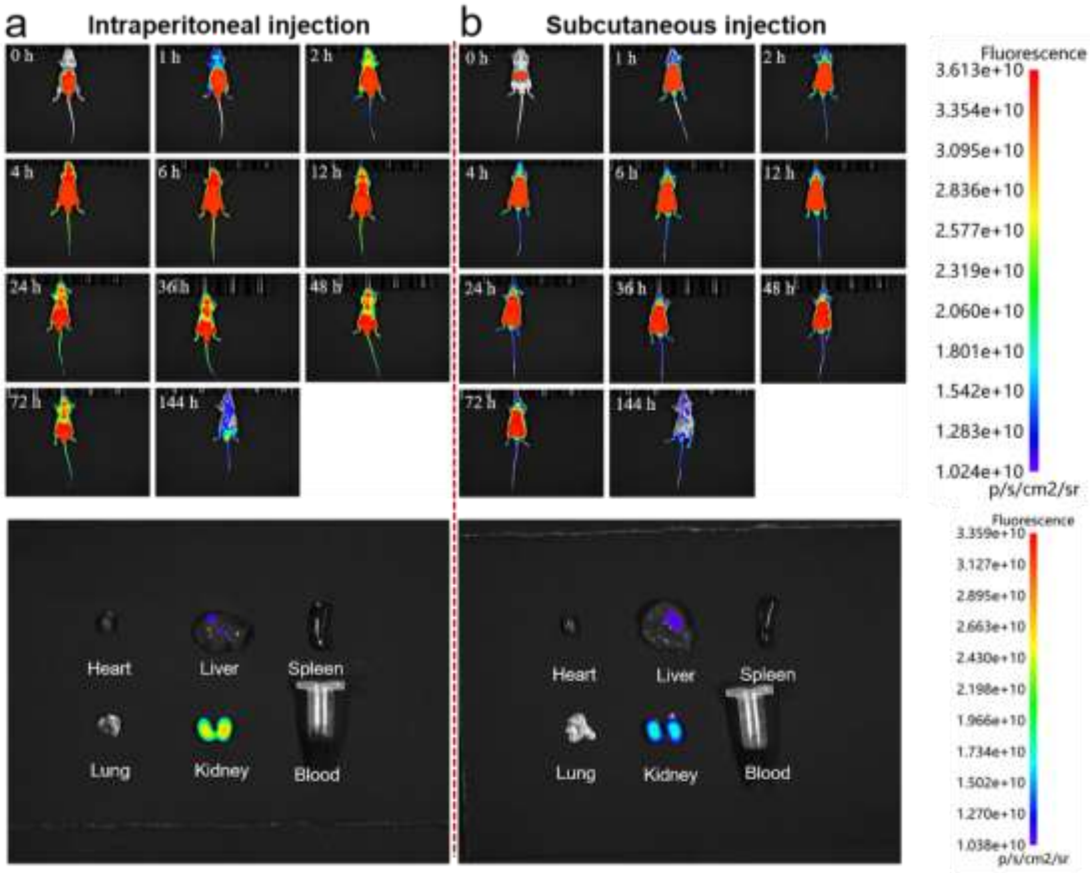

Fig. S26. In vivo distribution characteristics of fluorescently labeled PAA-CY7 in mice at different time points after subcutaneous injection and intraperitoneal injection to simulate the degradation process of the adhesive after application on subcutaneous tissues or surfaces of organs such as the liver. (a and b) Specifically, (a) shows the in vivo distribution of fluorescent PAA-CY7 at 0, 1, 2, 4, 6, 12, 24, 36, 48, 72, and 144 h post-intraperitoneal injection, while (b) shows that post-subcutaneous injection. It also includes representative fluorescence images of major organs (heart, liver, spleen, lungs, kidneys) and blood, which were obtained by dissecting the mice after 144 h. After the PAA-CY7 macromolecular chains were injected into the mice, they rapidly diffused throughout the body within 4-6 h. Following 6 days (144 h) of in vivo metabolism, the final fluorescence intensity decreased significantly or even disappeared completely, which was attributed to the excretion of the macromolecular chains from the body via physiological pathways. Analysis of the fluorescence intensity of major organs and blood at the end of the experiment revealed that almost no fluorescence signal was detected in the heart, spleen, lungs, or blood. The kidneys exhibited the highest fluorescence intensity, followed by the liver. This result indicates that the PAA-CY7 macromolecular chains (with a number-average molecular weight cut-off, M_n ~70 kDa) accumulate in the kidneys and are primarily excreted from the body through the renal pathway.

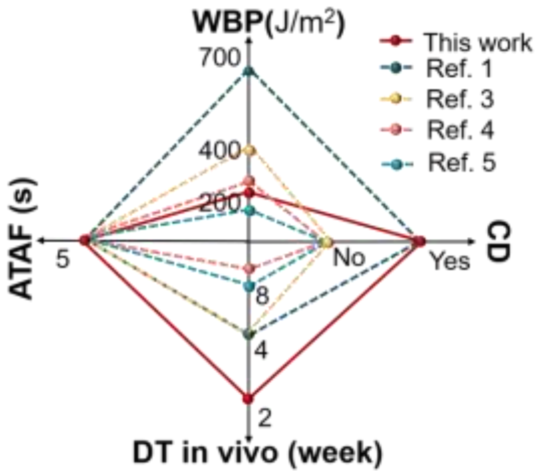

Fig. S27. Performance comparison of SeDH and other representative tissue adhesives. Comparison between SeDH and other representative tissue adhesives in terms of wet bioadhesion properties (WBP), application time for adhesion formation (ATAF), degradation time in vivo (DT in vivo), and research with controllable degradation (CD) or without.

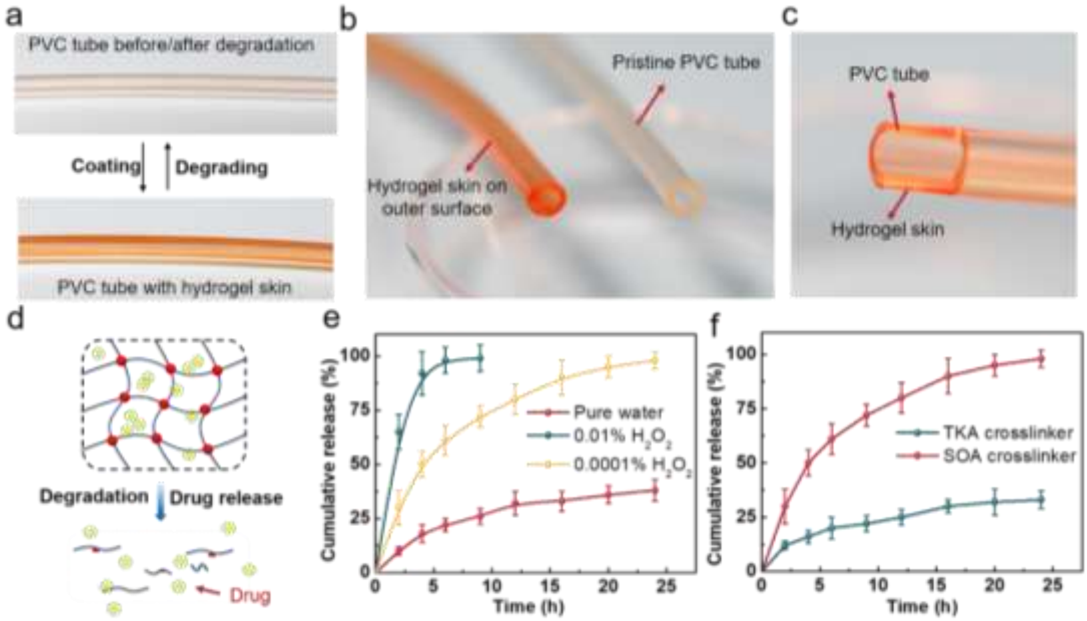

Fig. S28. ROS-responsive hydrogels in biomedical expansion applications. (a-c) PVC catheter coatings with rapidly degrading hydrogel skins, capable of reducing friction between the catheter wall and the tissue. (d-f) Drug delivery can be controlled by adjusting the proportion of degradable crosslinkers in the hydrogel, allowing for regulated drug release rates (37℃).

Table S1. Harmonic frequency analysis results for the TS1: frequencies and infrared intensities.

Mode	Frequency of TS1 (cm^{-1})	Frequency of TS2 (cm^{-1})
1	-631.22	-608
2	24.87	17.56
3	31.48	21.52
4	37.9	31.18
5	44.17	37.64
6	49.37	57.69
7	51.03	64.6
8	62.01	72.37
9	64.42	74.81
10	69.71	80.85
11	73.54	83.94
12	77.51	100.7
13	78.72	113.7
14	103.42	119.21
15	111.25	123.63
16	114.53	144.55
17	127.38	165.33
18	133.6	191.28
19	148.07	198.38
20	184.52	206.72
21	196.14	217.04
22	203.24	222.41
23	218.54	238.91
24	254.57	245.19
25	259.95	254.04
26	263.66	258.6
27	275.35	277.77
28	300.66	291.68
29	306.91	304.79
30	318.51	310.65
31	322.56	322.91
32	334.37	331.46
33	371.24	346.52
34	375.91	357.15
35	407.13	385.45
36	440.61	476.88
37	442.52	486.44
38	509.59	504.88
39	515.28	506.29

Mode	Frequency of TS1 (cm^{-1})	Frequency of TS2 (cm^{-1})
40	520.54	524.63
41	556.33	548.81
42	586.48	576.53
43	620.84	624.35
44	660.19	647.61
45	670.8	668.79
46	694.86	670.68
47	703.75	681.63
48	749.99	713.21
49	760.93	750.71
50	818.24	773.3
51	831.7	822.86
52	834.08	826.44
53	896.34	832.66
54	902.03	834.11
55	1015.13	895.18
56	1020.35	898.29
57	1025.06	1018.23
58	1026.16	1019.83
59	1027.42	1025.3
60	1030.49	1029.14
61	1044.32	1030.27
62	1049.24	1032.85
63	1052.39	1037.53
64	1064.95	1061.14
65	1083.21	1063.66
66	1089.46	1080.11
67	1100.12	1082.14
68	1108.87	1094.34
69	1216.43	1102.89
70	1222.85	1213.98
71	1242.99	1216.73
72	1254.54	1261.96
73	1267.57	1266.69
74	1278.49	1275.73
75	1340.62	1279.77
76	1343.95	1328.83
77	1351.6	1335.02
78	1353.14	1346.24
79	1399.49	1350.34

Mode	Frequency of TS1 (cm^{-1})	Frequency of TS2 (cm^{-1})
80	1411.44	1393.11
81	1418.77	1405.49
82	1436.13	1426.17
83	1437.55	1428.15
84	1450.91	1433.2
85	1465.34	1467.67
86	1470.04	1488.29
87	1474.06	1495.39
88	1481.43	1563.63
89	1563.99	1569.07
90	1568.54	1656.93
91	1679.05	1662.76
92	1679.86	1682.91
93	1731.18	1727.89
94	1732.3	1731.52
95	3113.1	3109.25
96	3121.16	3127.39
97	3122.78	3129.02
98	3130.18	3136.32
99	3169.92	3171.63
100	3171.88	3171.7
101	3172.4	3172.69
102	3179.3	3187.22
103	3187.95	3192.19
104	3203.88	3199.58
105	3206.45	3210.35
106	3207.13	3221.11
107	3267.69	3268.87
108	3269.42	3268.99
109	3582.69	3561.93
110	3629.39	3629.6
111	3678.21	3634.54
112	3803.94	3720.41
113	3872.6	3812.79
114	3877.49	3864.8

Table S2. Comparison of adhesion and biodegradation performance between SeDH and various recent representative tissue adhesives.

Tissue adhesives	Wet bioadhesion properties	Application time for adhesion formation/s	In vitro degradation time/days	In vivo degradation time /days	Research on controllable degradation
CST[1]	~710 J/m^2	5	~8	>28	√
ACPs[2]	~670 J/m^2	<10	/	/	/
PBAc/polyols films[3]	~400 J/m^2	5	>28	>28	/
ATGels[4]	~230 J/m^2	Instant	>28	>56	/
PAHN/PEGDA/PVA[5]	~160 J/m^2	5	>42	>28	/
NB-CMC/CMC hydrogel[6]	~100 J/m^2	/	>15	/	√
GmTAC[7]	~450 J/m^2	Instant	~14	/	/
GelMA/HA-NB[8]	~40 kPa	/	/	>56	/
HA-PEG/HA-SS-PEG[9]	~28 kPa	/	>10	35-175	√
Electro-Ox hydrogel tape[10]	~1200 J/m^2	1-21600	>>10	/	/
Fibrin glue	~5 kPa	180	/	~30 weeks	/
CPB[11]	~250 J/m^2	~1-2	/	/	/
PPBA-PVA[12]	~40 kPa	Instant	/	/	/
**This work**	~200 J/m^2	5	~0.1	~14	√

Note: “/” indicates cases not mentioned or not studied in the paper.

DST: Double-sided tape; ACPs: Adhesive cryogel particles; PBAc/polyols films: Phenylboronic acid, acrylic acid, polymerizable acrylate moieties and polyols; ATGels: Acid-tolerant hydrogels; PAHN: Free radical copolymerization of acrylic acid, 2-hydroxyethyl methacrylate and acrylic acid N-hydroxysuccinimide ester; PEGDA: Poly(ethylene glycol) diacrylate; PVA: Polyvinyl alcohol; NB-CMC/CMC hydrogel: Imine crosslinking-based photoresponsive chitosan hydrogel; GmTAC: Gradient modulus tissue adhesive composite; GelMA/HA-NB: Methacrylated gelatin/glycosaminoglycan hyaluronic acid; HA-PEG: HA-ADH/4aPEG-OPA; HA-SS-PEG: HA-DTPH/4aPEG-OPA; Electro-Ox hydrogel tape: Electro-oxidized alginate-dopa hydrogel; CPB: composite patch integrated with black phosphorus nanosheets; PPBA-PVA: poly(N,N-dimethylethylenediamine-g-3-bromomethylphenylboronic acid) phosphazene-polyvinyl alcohol.

References

1. Yuk H, Varela CE, Nabzdyk CS *et al.* Dry double-sided tape for adhesion of wet tissues and devices. *Nature* 2019; **575**: 169-74.
2. Xue Y, Chen M, Cao J *et al.* Adhesive cryogel particles for bridging confined and irregular tissue defects. *Military Med Res* 2023; **10**: 15.
3. Xue Y, Zhang J, Chen X *et al.* Trigger-detachable hydrogel adhesives for bioelectronic interfaces. *Adv Funct Mater* 2021; **31**: 2106446.
4. Chen X, Zhang J, Chen G *et al.* Hydrogel bioadhesives with extreme acid-tolerance for gastric perforation repairing. *Adv Funct Mater* 2022; **32**: 2202285.
5. Zhang K, Chen X, Xue Y *et al.* Tough hydrogel bioadhesives for sutureless wound sealing, hemostasis and biointerfaces. *Adv Funct Mater* 2022; **32**: 2111465.
6. Ma Y, Yao J, Liu Q *et al.* Liquid bandage harvests robust adhesive, hemostatic, and antibacterial performances as a first-aid tissue adhesive. *Adv Funct Mater* 2020; **30**: 2001820.
7. Li Y, Li G, Chen Y *et al.* Gradient modulus tissue adhesive composite for dynamic wound closure. *Adv Funct Mater* 2022; **32**: 2207306.
8. Hong Y, Zhou F, Hua Y *et al.* A strongly adhesive hemostatic hydrogel for the repair of arterial and heart bleeds. *Nat Commun* 2019; **10**: 2060.
9. Ren H, Zhang Z, Cheng X *et al.* Injectable, self-healing hydrogel adhesives with firm tissue adhesion and on-demand biodegradation for sutureless wound closure. *Sci Adv* 2023; **9**: eadh4327.
10. Xue B, Gu J, Li L *et al.* Hydrogel tapes for fault-tolerant strong wet adhesion. *Nat Commun* 2021; **12**: 7156.
11. Zhang Y, Li C, Guo A *et al*. Black phosphorus boosts wet-tissue adhesion of composite patches by enhancing water absorption and mechanical properties. *Nat Commun* 2024; **15**: 1618.
12. Ni Z, Yu H, Wang L *et al*. Polyphosphazene and non-catechol-based antibacterial injectable hydrogel for adhesion of wet tissues as wound dressing. *Adv Healthcare Mater* 2022; **11**: 2101421.

Movie S1.
Comparison of the degradable hydrogels in pure water and 0.1% H_2O_2 within 6 h (25℃).

Movie S2.
A wound on an ex vivo heart was sealed by SeDH adhesive.

Movie S3.
A wound on an ex vivo liver was sealed with SeDH adhesive.

Movie S4.
An irregular wound on an ex vivo liver was sealed by SeDH powder.

Movie S5.
Adhesive properties of SeDH powder.

Movie S6.
Peeling test of SeDH adhesive on different wet tissues.

Movie S7.
Lap shear test of SeDH adhesive on different wet tissues.

Movie S8.
Adhesive performance of SeDH on the moist dynamic heart.

Movie S9.
Hemostasis ability of SeDH adhesive.

Movie S10.
In vivo degradation performance of SeDH adhesive.
